# Predictive value of soluble suppression of tumorigenicity 2 in atrial fibrillation: a systematic review and meta-analysis

**DOI:** 10.3389/fcvm.2023.1308166

**Published:** 2024-01-11

**Authors:** Pengfei Chen, Jie Zhang, Jianpeng Du, Dazhuo Shi, He Zhang

**Affiliations:** ^1^ Xiyuan Hospital, China Academy of Chinese Medical Sciences, Beijing, China; ^2^ Cardiovascular Diseases Center, Xiyuan Hospital, China Academy of Chinese Medical Sciences, Beijing, China

**Keywords:** soluble suppression of tumorigenicity 2, atrial fibrillation, predictive, occurrence, recurrence, MACEs, meta-analysis, systematic review

## Abstract

**Purpose:**

Atrial fibrosis is the main pathological basis for the pathogenesis and progression of atrial fibrillation (AF). Soluble suppression of tumorigenicity 2 (sST2) is involved in fibrosis. Recent studies have explored its predictive value in AF outcomes. We performed this study to assess whether sST2 is an independent biomarker of AF outcomes and explore the potential mechanism.

**Methods:**

PubMed, Web of Science, EMBASE, and Cochrane Library databases were searched systematically from inception through July 1, 2023, to identify relevant studies. Outcomes of interest included occurrence, recurrence, and major adverse cardiac events (MACEs) of AF. This meta-analysis was reported following the criteria outlined in PRISMA 2020, and the protocol was registered in PROSPERO (number: CRD42023459789). All statistical analyses were performed using the STATA version 16.

**Result:**

Twenty four studies with 14,755 patients were included in the meta-analysis. The meta-analyses found that sST2 was significantly associated with the risk of occurrence [HR:1.04, 95% CI: 1.02–1.07, *P *< 0.01; *I*^2 ^= 67.8%], recurrence [HR:1.09, 95% CI: 1.02–1.16, *P *< 0.01; *I*^2^ = 89.5%], and MACEs (HR:1.60, 95% CI: 1.13–2.27, *P *< 0.01; *I*^2 ^= 82.0%) of AF. Furthermore, patients with AF showed higher sST2 than controls without AF (SMD: 0.41, 95% CI: 0.27–0.54, *P *< 0.01; *I*^2^*^ ^*= 0%), and AF patients with recurrence after catheter ablation (CA) showed significantly higher sST2 than those without recurrence (SMD: 0.81, 95% CI: 0.33–1.28, *P *< 0.01; *I*^2 ^= 83.9%). Sensitivity analyses showed that the outcomes were stable.

**Conclusions:**

Higher sST2 was association with an increased risk of occurrence, recurrence, and MACEs of AF. Assessing sST2 can be used as a potential screening method to predict AF outcomes.

**Systematic Review Registration:**

PROSPERO (CRD42023459789).

## 1 Introduction

Atrial fibrillation (AF) is one of the most common clinical arrhythmias, affecting more than 46.3 million individuals worldwide (1). The prevalence of AF is expected to double in the next 30 years to more than 17 million in Europe alone. AF leads to peripheral embolism, stroke, heart failure (HF), and is associated with high mortality and hospitalization rates (2). In addition, AF recurrence is also a challenging problem, with a recurrence rate of up to 30% (3). The pathophysiology of AF is complex and is thought to involve pro-inflammatory responses leading to electrophysiological remodeling, which in turn leads to atrial fibrosis and structural remodeling. The end result is to provide an arrhythmogenic substrate for AF triggers (4–6).

As with other diseases, blood markers have been used for the purpose of risk stratification for AF (7). The suppression of tumorigenicity 2 (ST2) is a member of the interleukin-1 (IL-1) receptor family, which exists in two forms: transmembrane receptor (ST2l) and soluble decoy receptor (sST2) (8). sST2 is relates to markers of hemodynamic load, released from the myocardium and vascular endothelial cells in response to pressure or volume overload, and is involved in fibrosis and remodeling through pathways related to inflammation (8, 9). sST2, mainly a well-known HF biomarker, and is also associated with worsening outcomes after myocardial infarction (MI) (10, 11). Recent studies have identified the predictive value of sST2 in AF (12–14). However, these studies are small and contradictory. Therefore, we aimed to assess whether sST2 is an independent biomarker of AF outcomes and explore the potential mechanism.

## 2 Methods

### 2.1 Search strategy

Two researchers performed a systematic literature search using four electronic databases (PubMed, Web of Science, EMBASE, and Cochrane Library) with MESH terms and keywords ((“Atrial fibrillation” OR “AF”) AND (“Biomarkers” OR “Soluble Suppression of Tumorigenicity 2” OR “Soluble ST2” OR “sST2” OR “ST2”). We also conducted a hand-searching of relevant articles. The disagreement was resolved by consulting a senior reviewer (Dazhuo Shi).

### 2.2 Literature inclusion and exclusion criteria

The inclusion criteria were: (1) study design was observational study (included prospective cohort, retrospective cohort, case-control, and cross-sectional study); (2) target population was AF patients; (3) there were measured sST2 at least two groups in one study; (4) outcomes of interest included occurrence, recurrence, and major adverse cardiac events (MACEs) of AF. The MACEs, which was defined as a composite outcome of fatal or non-fatal cardiovascular events, such as death, MI, HF, stroke, rehospitalization, and revascularization.

The exclusion criteria were: (1) abstracts, editorial, animal experiment, or review; (2) study with inadequate relevance; (3) study with insufficient clinical data.

### 2.3 Data extraction

The following data were extracted: (1) information on the publication: first author's name, publication year, location; (2) demographic characteristics: sample size, age, gender; (3) study details: study design, follow-up period, measurement methods of sST2, data on the diagnostic analysis (definition of the control group, sample size, mean ± standard deviatio (SDs) or median interquartile ranges (IQR) values, the optimal cut-off value, area under the curve (AUC) for the receiver operating characteristic curve (ROC), sensitivity, and specificity), and data on the prognostic analysis (clinical outcomes, unadjusted and/or multivariable-adjusted HRs/ORs, 95% CIs, the optimal cut-off value, AUC for the ROC, sensitivity, and specificity).

### 2.4 Bias assessment

The Newcastle-Ottawa Scale (NOS) (15) was used to assessed each study quality. NOS focused on three major aspects: participant selection (0–4 stars), comparability (0–2 stars), and exposure (0–3 stars). Studies were regarded as moderate-to-high quality with the total score ≥6, and <6 for low quality.

The Grading of Recommendations Assessment, Development and Evaluation (GRADE) (16) approach was used to assess the quality of outcome evidence. The quality were categorized into four levels (high, moderate, low, or very low). This meta-analysis only included observational studies, which start with a “low quality”, and other factors may then upgrade or downgrade the quality level.

### 2.5 Statistical analysis

All data of sST2 were pooled analysis by means ± SDs or HRs/ORs. The Cochrane Q-test and the *I*^2^-value were used to assess the statistical heterogeneity, where *P* < 0.1 or *I*^2^ > 50% suggested significant heterogeneity. A random-effect model were selected for this meta-analysis, considering the potential heterogeneity across studies. The publication bias was evaluated by employing the funnel plots and Egger's test, where a *P*-value higher than 0.1 indicated no significant publication bias. For sensitivity analysis, omitted one study at a time to assess the robustness. STATA version 16 was used for all statistical analyses.

## 3 Results

### 3.1 Study search

**Figure 1:**
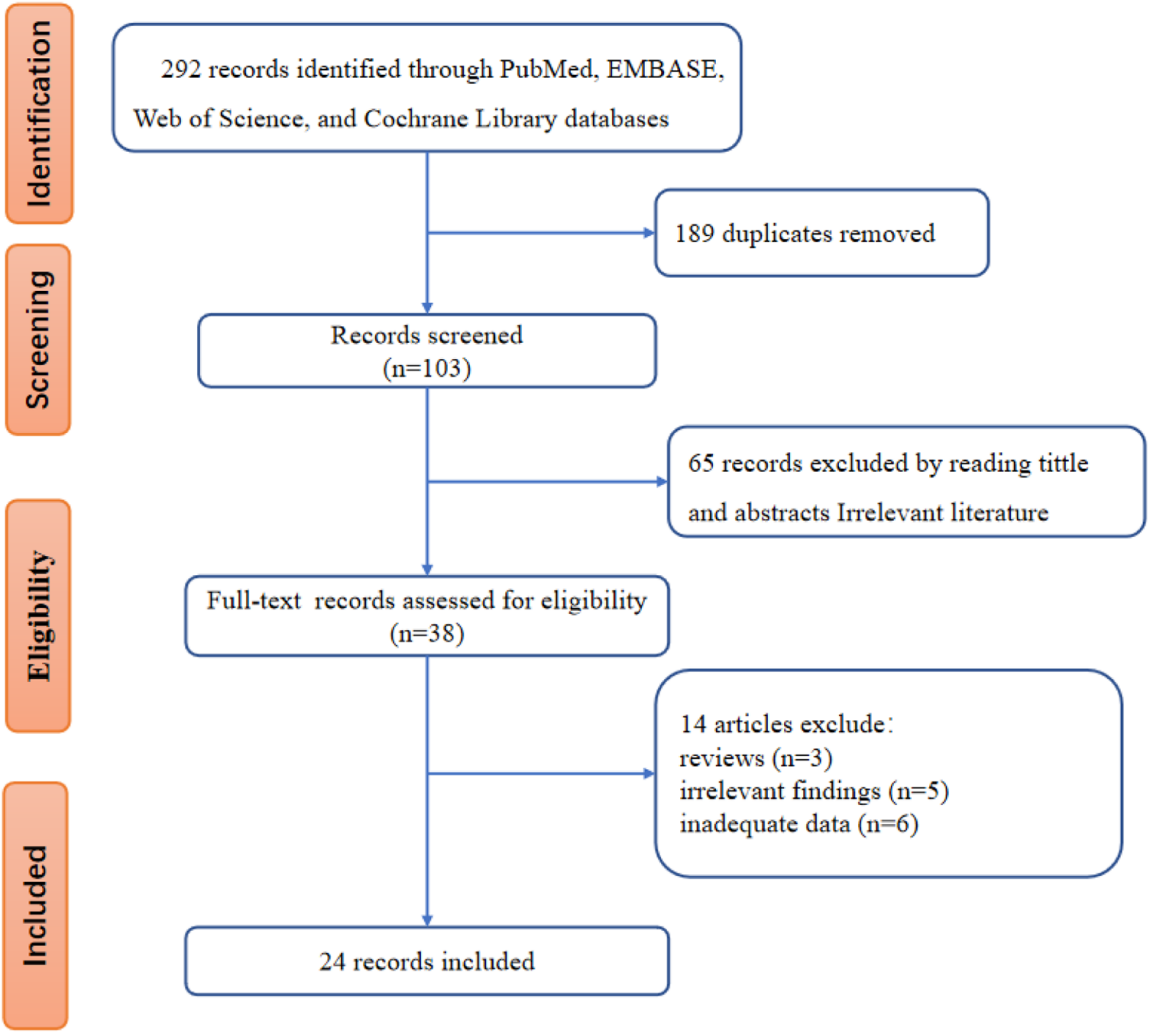
Flow chart of study selection and identification.

The flowchart of literature screening was shown in Figure 1. The initial database search identified 292 records, 189 of which were duplicates. According to the analysis of titles and abstracts, 65 studies were excluded, and 38 were included. After reading the full text, 14 studies were further excluded because 3 were reviews, 5 were irrelevant findings, and the other 6 were inadequate data. Finally, 24 studies (12–14, 17–37) were included.

### 3.2 Study characteristics

Table 1 displayed the baseline characteristics of 24 included studies from 2015 to 2022, comprising 14 prospective studies (14, 17–23, 26, 29–31, 33, 37), 4 retrospective studies (24, 25, 27, 32), 4 cross-sectional studies (12, 13, 34, 35), and 2 case-control studies (28, 36). Eleven studies were conducted in Asia (China, Thailand, Singapore), 10 studies in Europe (Germany, Poland, Finland, France, Norway, Spain, Sweden, Turkey), and 3 study in America (The united states). A total of 15,118 patients were involved, with an average of 62.07% males and an average age of 64.87 years. The follow-up time was 5.3–120 months. The included studies used various sources of sST2 reagents and adopted diverse detection strategies (e.g., 15 studies used ELISA to detect sST2, 2 used aspect plus assay, 1 used plasma samples assay, and 1 used duoset immunoassay assay). Concerning the purpose of these studies, 12 studies (12, 13, 21, 24, 25, 27, 28, 32, 34–37) evaluated the predictive value of sST2 in occurrence of AF, while 8 studies (14, 19, 20, 22, 26, 29, 31, 33) evaluated AF recurrence, 5 studies (17, 18, 23, 30, 37) evaluated MACEs following AF.

**Table 1:** Baseline characteristics of the 24 selected research.

Author (publication year)	Location	Study design	No. of patients	Age (years)	Men (%)	Follow-up (months)	Purpose	NOS	Source of sST2 assay kit	sST2 detection method and unit
Ana Merino-Merin 2022	Spain	Cross-sectional	148	62.5	66.9	–	Diagnostic	7	–	ng/ml
Are A. Kalstad 2021	Norway	Cross-sectional	299	75	70.2	–	Diagnostic	7	Critical Diagnostics, San Diego, US	ELISA ng/ml
Bi-Xi Chen 2021	China	Prospective cohort	70	66	64	27	Prognostic	8	R&D Systems Inc, Minneapolis, MN	ELISA ng/ml
Chang-Xi Chen 2018	China	Prospective cohort	290	64.6	63.1	6	Prognostic	7	Boyun, Shanghai, China	ELISA ng/L
Eugene S.J. Tan 2020	Singapore	Prospective cohort	261	66.7	73	24	Prognostic	8	Critical Diagnostics, San Diego, US	ELISA ug/L
Hai-lei Liu 2020	China	Prospective cohort	258	60.9	56.6	13.5	Prognostic	8	Critical Diagnostics, San Diego, CA, USA	ELISA ng/ml
Jan Budzianowski 2021	Poland	Prospective cohort	114	62.3	52.6	24	Prognostic	9	R&D Systems, Minneapolis, MN, USA	DuoSet immunoassay ng/ml
Jan-Thorben Sieweke 2020	Germany	Prospective cohort	81	65	69.1	–	Diagnostic	7	Critical Diagnostics, San Diego, USA	ELISA ng/ml
Jia-li Fan 2022	China	Prospective cohort	84	64.2	57.1	12	Prognostic	8	ColorfulGene Biological Technology, Wuhan, China	ELISA pg/ml
Juan A. Vilchez 2015	Spain	Prospective cohort	562	77	49	48	Prognostic	8	Critical Diagnostics, San Diego, SA, USA	ELISA ug/L
Julio A. Lamprea-Montealegre 2019	Amerian	Retrospective cohort	3,053	57.2	53.8	96	Diagnostic	7	Critical Diagnostics, San Diego, SA, USA	ELISA ng/ml
Lei Chen 2022	China	Retrospective cohort	1,517	80	74	–	Diagnostic	6	Elabscience Biotechnology, China	ELISA ng/ml
Marc Badoz 2021	France	Prospective cohort	105	63	74.2	12	Prognostic	8	Eurobio Ingen (Les Ulis, France)	Aspect Plus ng/ml
Michiel Rienstra 2015	Amerian	Retrospective cohort	3,217	59	46	120	Diagnostic	8	Critical Diagnostics, San Diego, US	ELISA ng/ml
Nisha Bansal 2022	Amerian	Case-control	774	59.4	56	5.3	Diagnostic	7	R&D Systems, Minneapolis, MN, USA	ELISA ng/ml
Paweł Wałek 2020	Poland	Prospective cohort	80	64.7	70	12	Prognostic	8	Critical Diagnostics, San Diego, CA, USA	ELISA ng/ml
Rungroj Krittayaphong 2022	Thailand	Prospective cohort	185	68.9	62.7	33.1	Prognostic	8	Critical Diagnostics, San Diego, SA, USA	plasma samples ng/ml
Ruopeng Tan 2021	China	Prospective cohort	210	58.2	68.1	15	Prognostic	8	Critical Diagnostics, San Diego, CA, USA	ELISA ng/ml
Santeri Nortamo 2017	Finland	Retrospective cohort	1,710	68	64	60	Diagnostic	8	R&D Systems Inc, Minneapolis, MN	ELISA ng/ml
Sefa Okar 2018	Turkey	Prospective cohort	100	55.1	47	12	Prognostic	9	Critical Diagnostics, San Diego, CA, USA	Aspect Plus ng/ml
Wei-Ping Sun (a) 2022	China	Cross-sectional	359	57	61	–	Diagnostic	6	–	–
Wei-Ping Sun (b) 2022	China	Cross-sectional	181	52.9	67.6	–	Diagnostic	7	–	pg/ml
Xian-liang Yan 2022	China	Case-control	306	74.2	53.6	–	Diagnostic	7	–	–
Zainu Nezami 2022	Sweden	Prospective cohort	316	75	70	6	Diagnostic, Prognostic	7	Olink Bioscience, Uppsala, Sweden	Proximity extension assay

For complete study names, see Reference.

### 3.3 Study quality

The mean NOS scores was 7.54 (range 6–9), indicating moderate to high quality. The details of the quality assessment were shown in Supplementary Table S1.

The GRADE grade showed that 2 evidences were mediate, 1 was low, and 2 were very low. The results of the GRADE assessment were shown in Supplementary Table S2.

### 3.4 Results of meta-analysis

#### 3.4.1 Predictive value of sST2 in occurrence of AF

**Figure 2:**
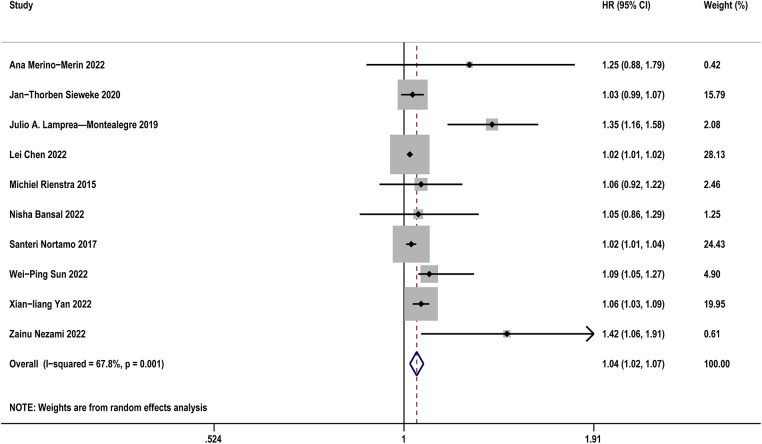
Forest plots show the relationship between sST2 (continuous variables) and the risk of AF occurrence.

Table 2 diaplayed the characteristics of the included studies for predictive value of sST2 in occurrence of AF. A total of 12 studies were analyzed with 11,961 participants (including 1,707 AF patients and 10,254 controls). The pooled analysis of 10 studies (12, 21, 24, 25, 27, 28, 32, 34, 36, 37) as a continuous variable indicated that sST2 was associated with the risk of AF occurrence (HR:1.04, 95% CI: 1.02–1.07, *P *< 0.01; *I*^2 ^= 67.8% Figure 2). Sensitivity analyses showed that the outcomes were stable (Supplementary Figure S2A). The funnel plot and Egger's test revealed publication bias in the results (Egger's test, *P* = 0.006, Supplementary Figures S2B,C).

**Table 2:** Characteristics of the 12 studies for predictive value of sST2 in occurrence of AF.

Author (publication year)	No. of AF group patients	sST2 of AF patients, mean (SD)/median (IQR)	Definition of the control group	No. of control group patients	sST2 of controls, mean (SD)/median (IQR)	Univariable analysis	Multivariable analysis	AUC	Cutoff Value	Sensitivity (%)	Specificity (%)
Ana Merino-Merin 2022	115	35.43 ± 15.89	Healthy controls	33	27.43 ± 10.95		1.25 (0.88,1.79)	0.648	37.7	40%	82%
Are A. Kalstad 2021	38	31.6 (25.1, 39.7)	MI	261	28.6 (23.4, 34.3)	4.13 (1.69–10.13)	4.31 (1.61,11.61)	0.608 (0.508,0.707)			
Jan-Thorben Sieweke 2020	21	29.3 ± 10.1	Healthy controls	60	24.9 ± 12.8	1.03 (0.99,1.07)					
Julio A. Lamprea-Montealegre 2019	279		CKD	2,774			1.35 (1.16,1.58)				
Lei Chen 2022	115	55.1 (39.1–97.95)	MI	1,402	30.1 (23.9,40.5)	1.02 (1.015,1.024)	1.02 (1.014,1.02)	0.827 (0.790,0.864)	35.15	89.6%	65.3%
Michiel Rienstra 2015	242		Healthy controls	2,975			1.06 (0.92,1.22)				
Nisha Bansal 2022	123		CKD	651			1.05 (0.86,1.29)				
Santeri Nortamo 2017	143	20.2 ± 10.8	CHD	1,567	17.5 ± 7.2	1.037 (1.020,1.055)	1.025 (1.007,1.043)				
Wei-Ping Sun (a) 2022	249	14.0 (10.4,20.8)	Healthy controls	110	9.1 (6.7,12.4)		1.086 (1.046,1.272)	0.7954 (0.813–0.922)	12.81	78.4%	75.4%
Wei-Ping Sun (b) 2022	68	12.76 ± 4.73	Healthy controls	113	10.3 ± 4.82						
Xian-liang Yan 2022	120	40.6 (25.9,53.6)	HF	186	23.7 (16.3,35.9)		1.06 (1.03,1.09)				
Zainu Nezami 2022	194	5.71 (0.86)	HF	122	5.41 (0.99)	1.40 (1.10,1.78)	1.42 (1.06,1.91)				

SD, standard deviation; IQR, interquartile range; MI, myocardial infarction; CKD, chronic kidney disease; CHD, coronary heart disease; HF, heart failure; AF, atrial fibrillation.

**Figure 3:**
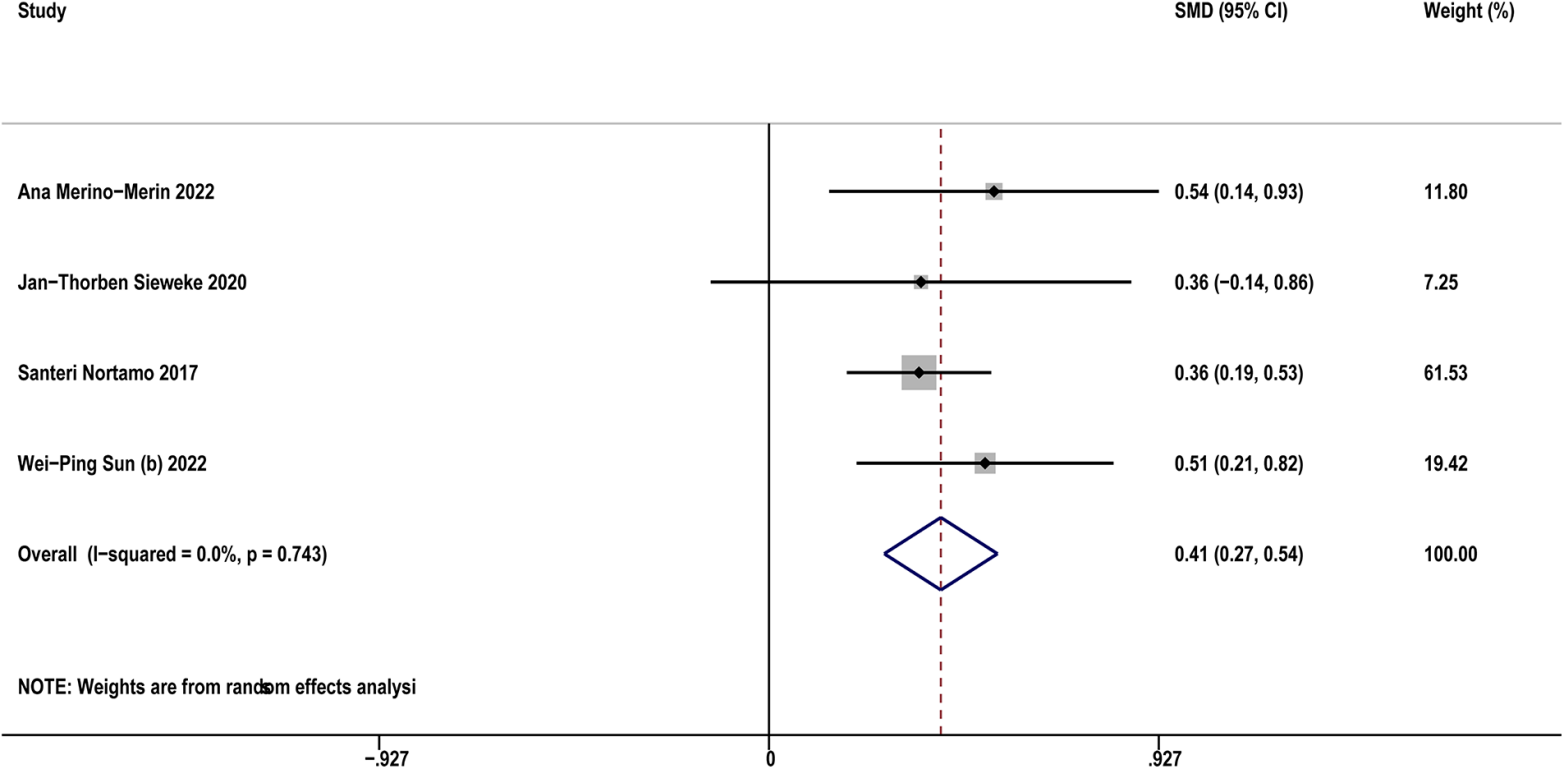
Forest plots show the difference in sST2 values between patients with and without AF.

Four studies (12, 21, 32, 35) compared sST2 between AF patients and controls. The result indicated that AF patients showed higher sST2 than controls (SMD: 0.41, 95% CI: 0.27–0.54, *P *< 0.01; *I*^2^*^ ^*= 0% Figure 3). Sensitivity analyses found that the outcomes were stable (Supplementary Figure S3A). Publication bias was not indicated (Egger's test, *P* = 0.398, Supplementary Figures S3B,C).

#### 3.4.2 Predictive value of sST2 in AF recurrence after CA

**Figure 4:**
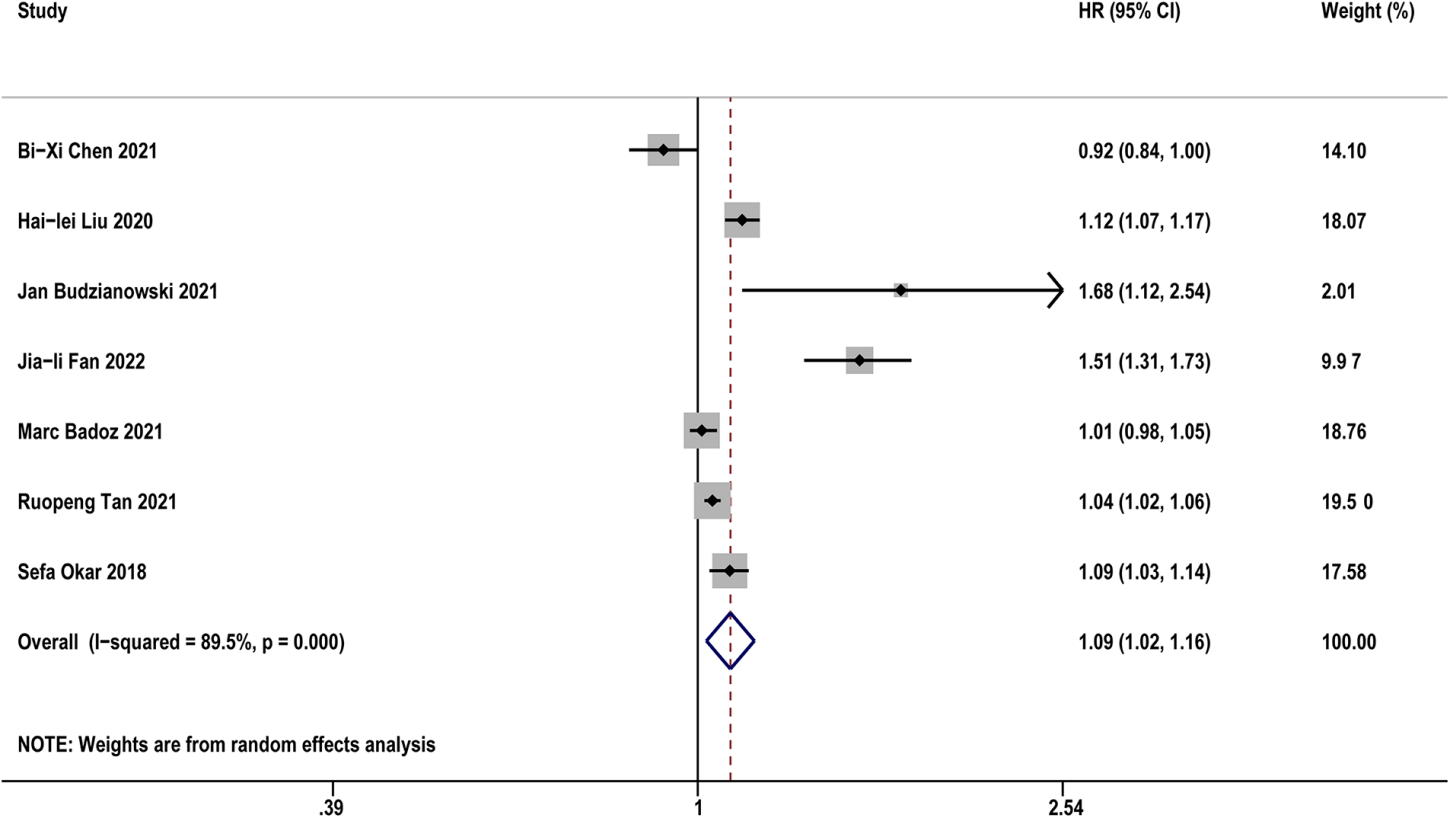
Forest plots show the relationship between sST2 (continuous variables) and the risk of AF recurrence after CA.

Table 3 diaplayed the characteristics of the included studies for predictive value of sST2 in recurrence of AF. A total of 8 studies were analyzed with 1,021 AF patients (including 255 AF recurrence and 766 not recurrence). The pooled analysis of 7 studies (14, 19, 20, 22, 26, 31, 33) as a continuous variable indicated that sST2 was associated with the risk of AF recurrence (HR:1.09, 95% CI: 1.02–1.16, *P *< 0.01; *I*^2 ^= 89.5% Figure 4). Sensitivity analyses showed that the outcomes were stable (Supplementary Figure S4A). Publication bias was not indicated (Egger's test, *P* = 0.249, Supplementary Figures S4B,C).

**Table 3:** Characteristics of the 8 studies for prognostic analysis of AF recurrence after CA.

Author (publication year)	No. of AF patients	No. of AF recurrence	Univariable analysis	Multivariable analysis	sST2 of recurrence, mean (SD)/median (IQR)	sST2 of not recurrence,mean (SD)/median (IQR)	AUC	Cutoff Value	Sensitivity (%)	Specificity (%)
Bi-Xi Chen 2021	70	14		0.916 (0.839, 1.001)	13.6 ± 7.2	13.1 ± 6.5				
Hai-lei Liu 2020	258	52	1.015 (1.009, 1.021)	1.120 (1.072, 1.171)	31.3 ± 15.6	20.3 ± 7.1	0.948	26.9	100%	26.9%
Jan Budzianowski 2021	114	32		1.68 (1.115, 2.536)						
Jia-li Fan 2022	84	11	1.512 (1.312,1.726)				0.86 (0.77, 0.96)	59.17	89.5%	75.6%
Marc Badoz 2021	105	34		1.01 (0.98, 1.05)	30.3 (23.3, 39.8)	23.4 (17.4, 33.0)	0.678	26.7	73.5%	64.8%
Paweł Wałek 2020	80	47		1.08 (1.02, 1.13)	28 ± 22.9	16.9 ± 9.8	0.752 (0.634,0.870)	15.314	66.7%	85.1%
Ruopeng Tan 2021	210	43		1.038 (1.017, 1.06)	38.65 ± 17.78	32.44 ± 10.34	0.748	39.25	74%	77%
Sefa Okar 2018	100	22		1.085 (1.455, 3.040)	54.6 ± 35.1	22.8 ± 11.5	0.831	30.6	77.3%	79.5%

**Figure 5:**
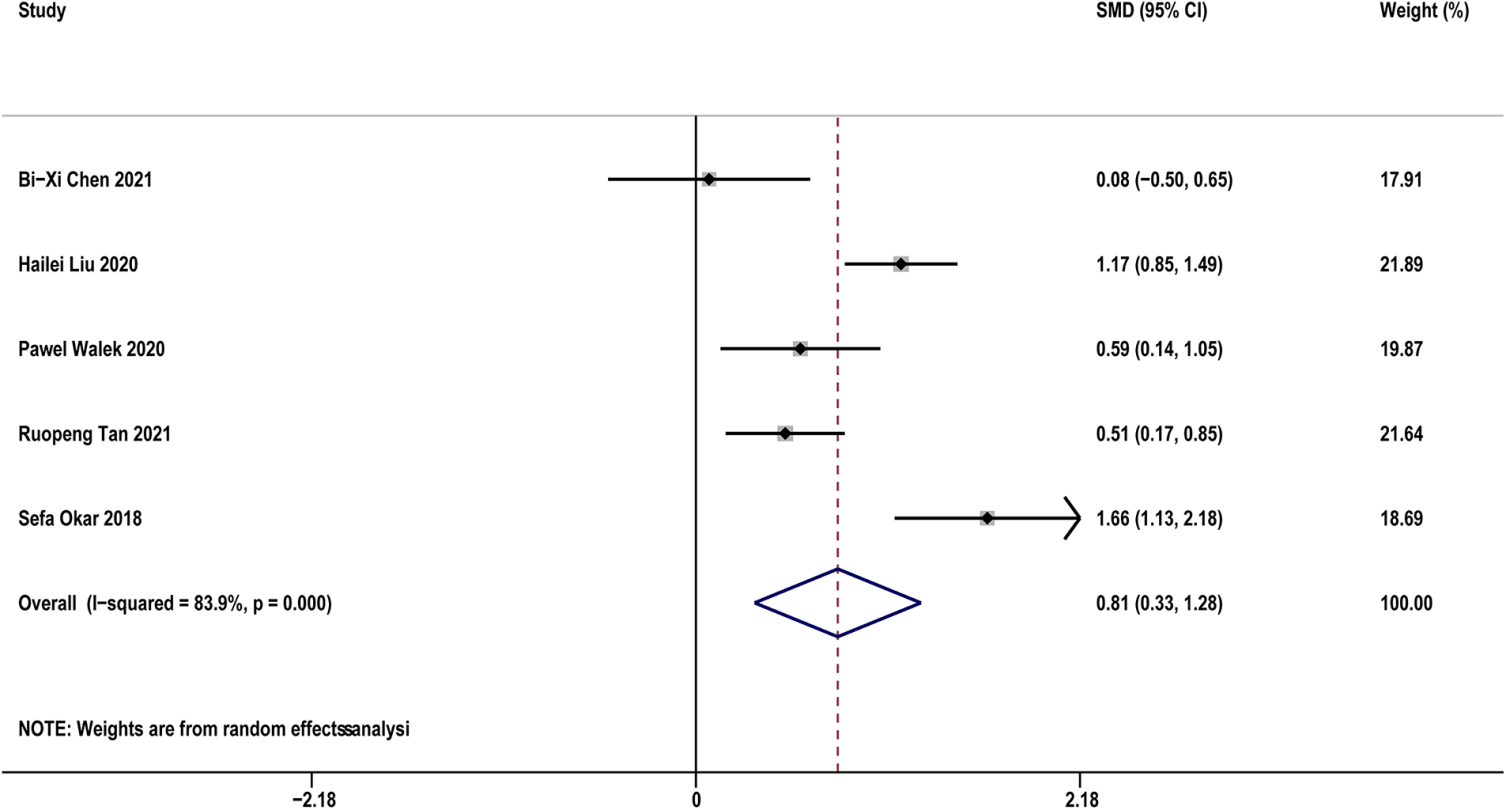
Forest plots show the difference in sST2 values between patients with and without AF recurrence after CA.

Five studies (14, 19, 29, 31, 33) compared sST2 between recurrence and not recurrence in AF patients. The results indicated that AF patients with recurrence showed significantly higher sST2 than those without recurrence (SMD: 0.81, 95% CI: 0.33–1.28, *P *< 0.01; *I*^2 ^= 83.9% Figure 5). Sensitivity analyses found that the outcomes were stable (Supplementary Figure S5A). Publication bias was not indicated (Egger's test, *P* = 0.832, Supplementary Figures S5B,C).

#### 3.4.3 Predictive value of sST2 in MACEs following AF

**Figure 6:**
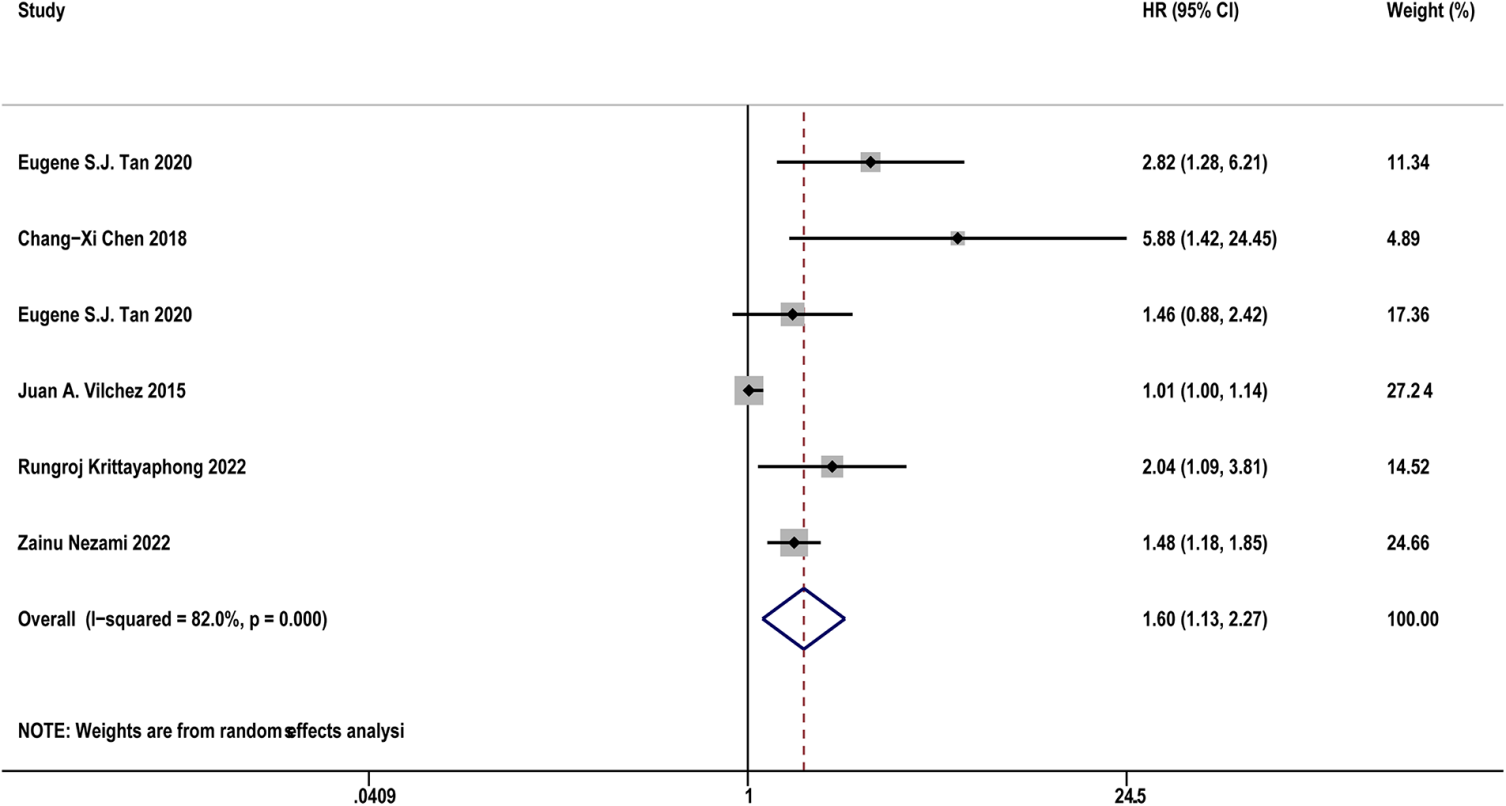
Forest plots show the relationship between sST2 (continuous variables) and the risk of MACEs following AF.

Table 4 diaplayed the characteristics of the included studies for predictive value of sST2 in MACEs following AF. Five studies (17, 18, 23, 30, 37) with 1,614 AF patients examined the relationship between sST2 and the risk of MACEs following AF. The pooled analysis of the estimates as a continuous variable indicated that sST2 was significantly associated with the risk of MACEs (HR:1.60, 95% CI: 1.13–2.27, *P *< 0.01; *I*^2 ^= 82.0% Figure 6). Sensitivity analyses showed that the outcomes were stable (Supplementary Figure S6A). The funnel plot and Egger's test revealed publication bias in the results (Egger's test, *P* = 0.004, Supplementary Figures S6B,C).

**Table 4:** Characteristics of the 5 studies for prognostic analysis of MACEs after AF.

Author (publication year)	Clinical outcome	Univariable analysis	Multivariable analysis	sST2 of patients with AF, mean (SD)/median (IQR)	sST2 sST2 of patients without AF, mean (SD)/median (IQR)	AUC	Cutoff Value	Sensitivity (%)	Specificity (%)
Chang-Xi Chen 2018	HF occurrence		5.88 (1.42, 24.45)			0.60 (0.51, 0.69)	219.3	95.4%	34.5%
Eugene S.J. Tan 2020	All-cause mortality		2.82 (1.28, 6.21)						
Eugene S.J. Tan 2020	First HF-hospitalization		1.46 (0.88, 2.42)						
Juan A. Vilchez 2015	all-cause mortality	1.77 (1.09, 2.86)	1.01 (1.002, 1.14)	1.66 (1.02, 2.70)			45.8	72%	42%
Rungroj Krittayaphong 2022	HF or death	3.25 (1.87, 5.65)	2.04 (1.09, 3.81)	54 39.8 ± 22.3	131 27.8 ± 17.4	0.69	30.14		
Zainu Nezami 2022	mortality	1.44 (1.20, 1.74)	1.48 (1.18, 1.85)						

## 4 Discussion

Our study found that sST2 was associated with the risk of occurrence, recurrence, and MACEs of AF. For every 1 unit increase in sST2, the risk of occurrence, recurrence, and MACEs increased by 4%, 9%, and 60%, respectively. Furthermore, patients with AF showed higher sST2 than controls without AF, and AF patients with recurrence showed significantly higher sST2 than those without recurrence. Sensitivity analyses showed that the outcomes were stable.

AF is partially explained by atrial remodeling determined by myocardial hypertension, dilatation, infiltration, inflammation and fibrosis (38, 39). Atrial remodeling acts in concert with the arrhythmia itself to enhance atrial vulnerability to AF, and might be both cause and consequence of AF (40, 41). Atrial fibrosis is a defining structural feature of atrial remodeling, a process that includes extracellular matrix (ECM) and collagen deposition accumulation and is thought to be initiated and regulated by immune cells (42, 43). Identifying biomarkers associated with atrial fibrosis will increase our understanding of the pathophysiological mechanisms of AF and could be used to develop pharmacological pathways for the prevention of AF, in addition, adding these biomarkers to the risk scale may lead to more accurate predictions of AF risk (44). sST2, as a biomarker closely related to fibrosis and inflammation, plays an important role in the pathogenesis and progression of AF, and also has certain value in predicting the occurrence, progression, recurrence and prognosis of AF.

Studies have found that sST2 reflect fibrosis may be related to the inhibition of IL-33/ST2l pathway (45, 46, 9). IL-33 is the functional ligand of ST2l, and the IL-33/ST2l signaling pathway has been shown plays a role in anti-myocardial fibrosis and cardiomyocyte hypertrophy (47, 48). sST2 is a receptor for IL-1 and competitively binds to IL-33 to inhibit the protective effect of IL-33/ST2l on myocardium (49). When myocardium induced by pressure or volume overload produce a large amount of sST2, high concentrations of sST2 prevent IL-33/ST2l effects and may therefore lead to atrial fibrosis, which is associated with AF via atrial structural remodeling (49, 50). Early atrial dilation in AF patients can lead to physiological stretching of the atrium, causes myofibroblasts to release IL-33, which binds ST2l to myocardial cell membranes and promotes cell integrity and survival. However, during long-term lesions, local and adjacent cells can increase the release of the IL-33 decoy sST2, thereby blocking IL-33/ST2l binding and promoting tissue fibrosis (51, 52). Notably, the novel aspect of IL-33/ST2l signaling mediating cardiac fibrosis represents some novel biomolecular targets for prevention and treatment of AF. In addition, sST2 can also cause myocardial damage by promoting oxidative stress and inflammation. sST2 affects mitochondrial fusion of human cardiac fibroblasts and increases oxidative stress production and inflammatory marker secretion by reducing mitofusins-1 (MFN-1) expression (53).

Fibrosis is associated with impaired cellular coupling, enhanced heterogeneity of intra-atrial conduction, and dispersion of refractory periods, which provide substrates for sustained reentry to drive AF (54, 43). We think that sST2 can reflect fibrosis as well as the degree of fibrosis. Studies have found that the expression of sST2 in patients with persistent AF is higher than that in patients with paroxysmal AF, which may explain that sST2 reflects the degree of fibrosis and the progression of AF (55, 56). Recurrence after AF ablation is directly related to atrial fibrosis and is positively correlated (57). Our study found that sST2 level is an independent predictor of AF recurrence after CA, and every 1 unit increase in sST2, the risk of recurrence increased by 9%. sST2 is also directly associated with MACEs of AF patients. Increased abnormal hemodynamic load leads to atrial dilation, which is a well-known cause of the development of AF, and may also stimulate sST2 and BNP secretion. In addition, sST2 levels are elevated during AF, possibly because the heart rate and atrial pressure in patients with AF are higher than normal, thereby increasing cardiac burden and increasing the risk of MACEs (58, 59). IL-33/ST2l signaling is thought to play an important role in regulating the myocardial response of stretched cardiac fibroblasts and cardiomyocytes to biomechanical overload (60). Loss of IL-33/ST2l signaling leads to hypertrophy of cardiomyocytes, fibrosis, and deterioration of left ventricular function, further aggravating ventricular myocardial remodeling, and increasing the risk of death from HF. Our results show that sST2 has a stronger correlation with the risk of MACEs (HR = 1.60) than the occurrence (HR = 1.04) and recurrence (HR = 1.09) of AF, indicating that sST2 has notable prognostic performance, but low diagnostic performance. We believe that sST2, as a new biomarker of inflammation, fibrosis and cardiac stress, may have a more direct correlation associated with cardiac damage and MACEs. And the prognostic information provided by sST2 is in addition to that provided by other well stablished biomarkers, such as BNP and troponins. However, the mechanism of AF occurrence and recurrence is complex, and sST2, although a marker of fibrosis, is not specific to atrial fibrosis, thus showing a weak clinical correlation.

As a new biomarker, galectin-3 (Gal-3) and sST2 have been found to play an important role in fibrosis. Gal-3 can specifically bind to ECM and fibroblasts, thereby promoting fibroblast proliferation, inflammatory cell infiltration, and remodeling (57). However, the current research is not very clear about the mechanism of Gal-3 and sST2 on atrial fibrosis, and future research should be performed to reveal their role and mechanism, and provide more new basis for the prevention and treatment of atrial fibrosis.

## 5 Strengths and limitations

Our study performed a detailed meta-analysis and mechanism analysis. First, this is the first meta-analysis to summarize the predictive value of sST2 in AF. Second, this study covers a large sample size from different countries, so the results are relatively stable and reliable. Third, the pooled analysis was based on the most adequately adjusted HRs, so the finding may be independent of potential confounders. Fourth, sensitivity analyses did not significantly affect the results, indicating the results were credible.

There were some limitations in our study. First, the meta-analysis only included observational studies, it carries inherent study design limitations. Second, the heterogeneity in meta-analyses were significant. We used sensitivity analysis and publication bias to explore the source of heterogeneity. Third, some residual factors may affect the results. Fourth, we did not find some suitable case reports to prove our study.

## 6 Conclusions

Higher sST2 was association with an increased risk of occurrence, recurrence, and MACEs in AF. Assessing sST2 can be used as a potential screening method to predict AF outcomes. Further well-designed cohort studies and randomized clinical trials are warranted to confirm this finding.

## Data Availability

The original contributions presented in the study are included in the article/Supplementary Material, further inquiries can be directed to the corresponding authors.
