# Supplementary figures and images for: Predictive value of soluble suppression of tumorigenicity 2 in atrial fibrillation: a systematic review and meta-analysis
Source: Front Cardiovasc Med. 2024 Jan 11;10:1308166. doi: 10.3389/fcvm.2023.1308166 (PMC10808625; doi:10.3389/fcvm.2023.1308166)

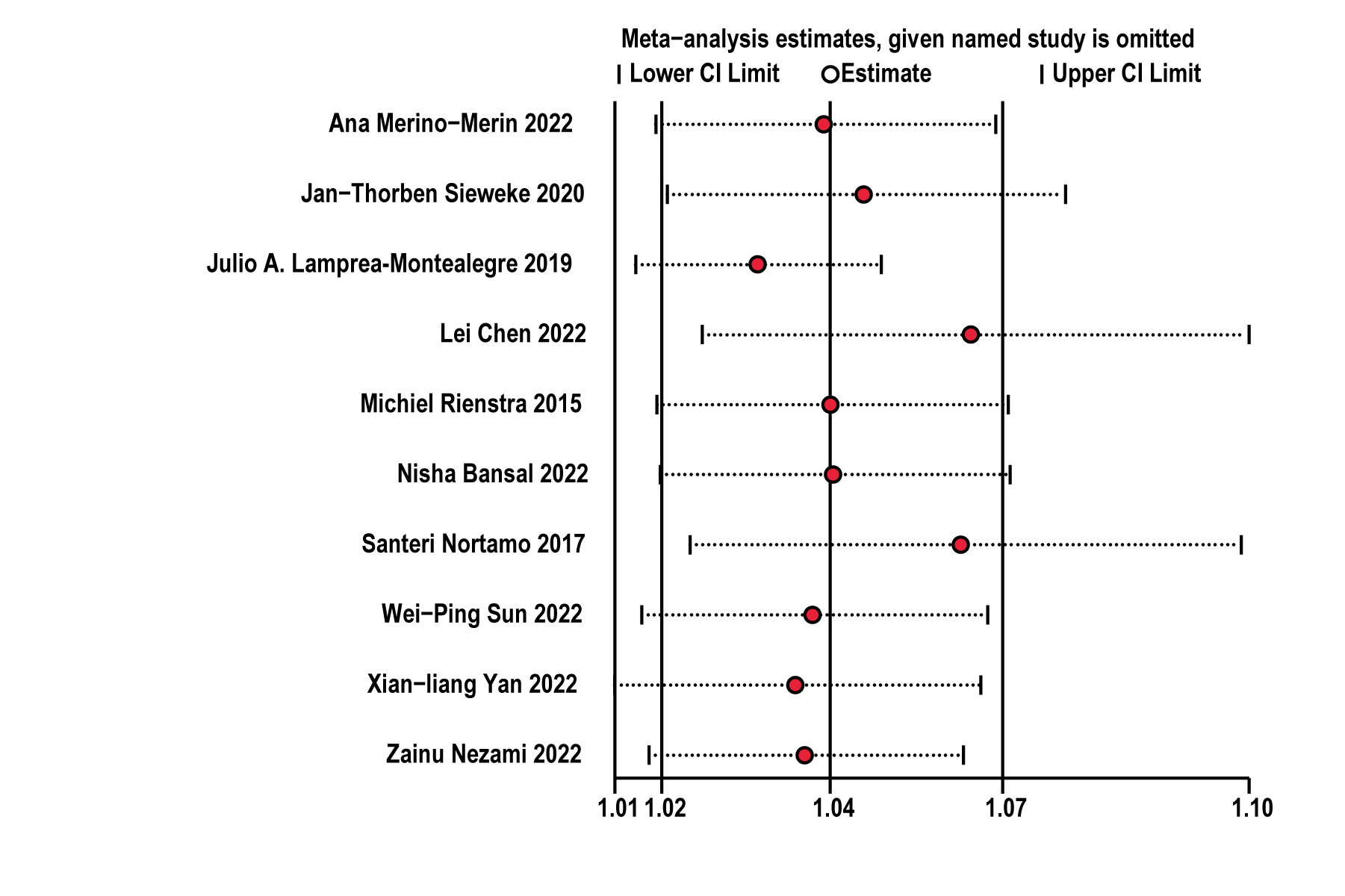

Supplement: Supplementary file 1 [file Datasheet1.zip › Data Sheet 1_v1/Supplementary Figure2A.tif]

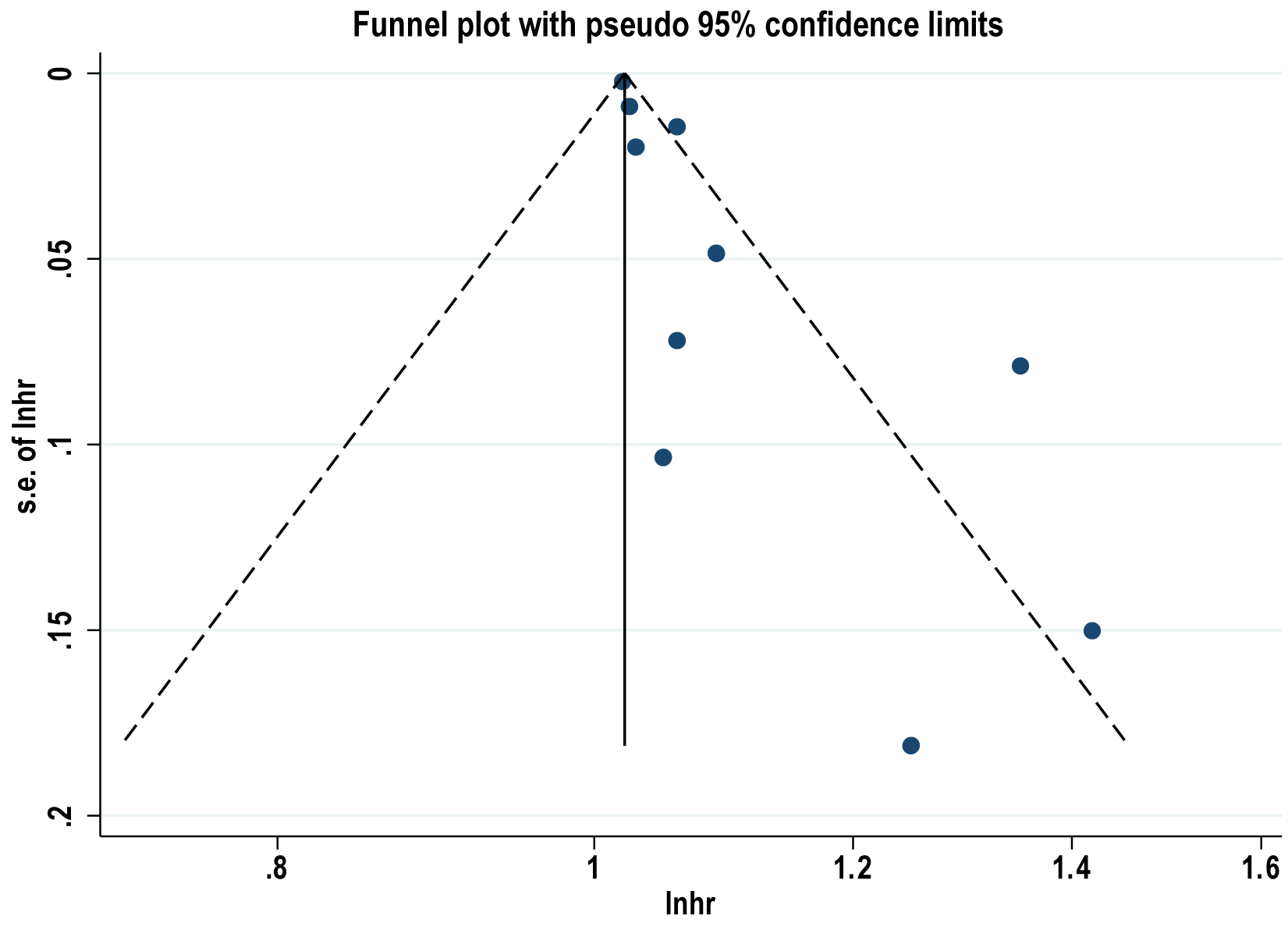

Supplement: Supplementary file 1 [file Datasheet1.zip › Data Sheet 1_v1/Supplementary Figure2B.tif]

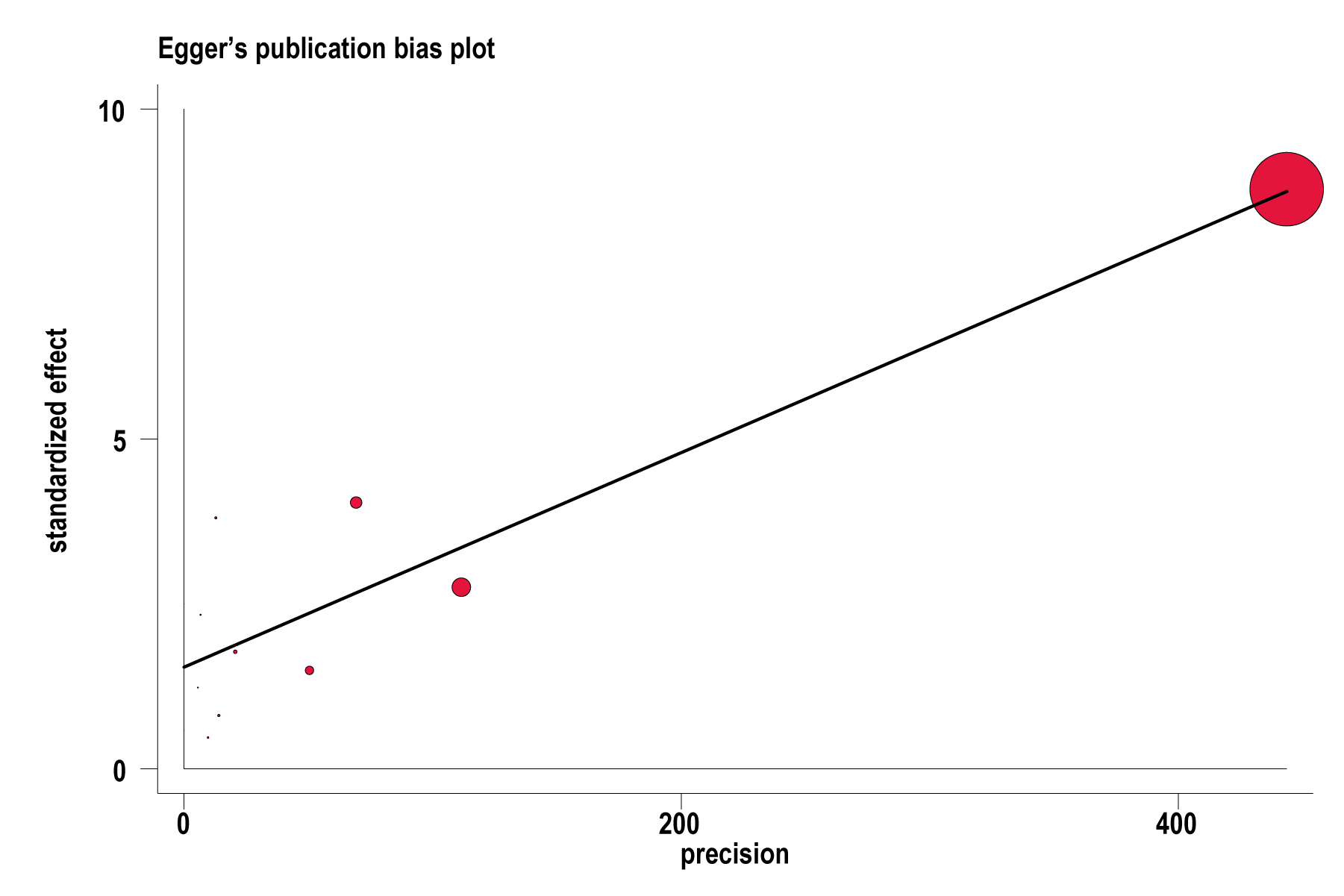

Supplement: Supplementary file 1 [file Datasheet1.zip › Data Sheet 1_v1/Supplementary Figure2C.tif]

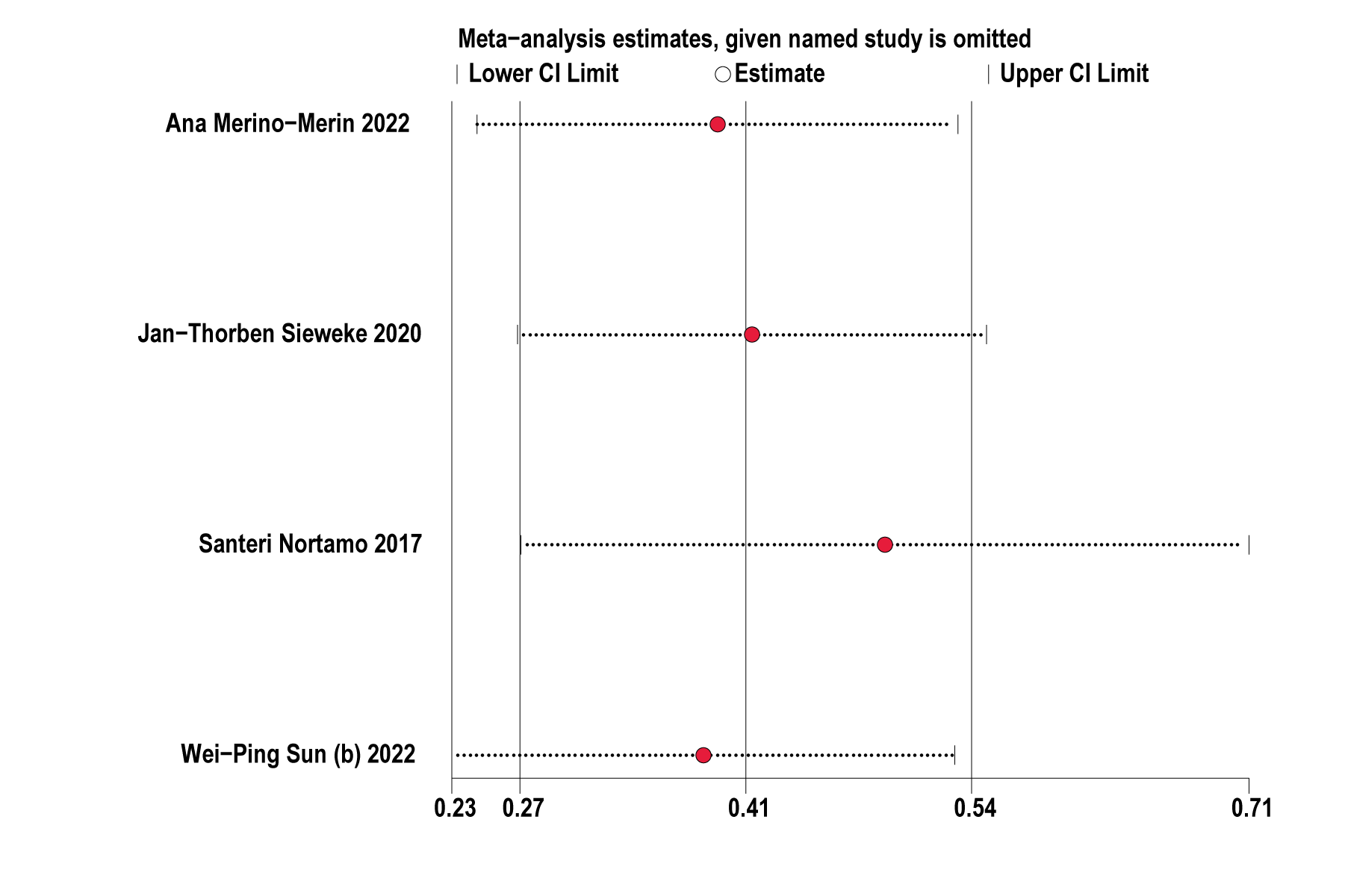

Supplement: Supplementary file 1 [file Datasheet1.zip › Data Sheet 1_v1/Supplementary Figure3A.tif]

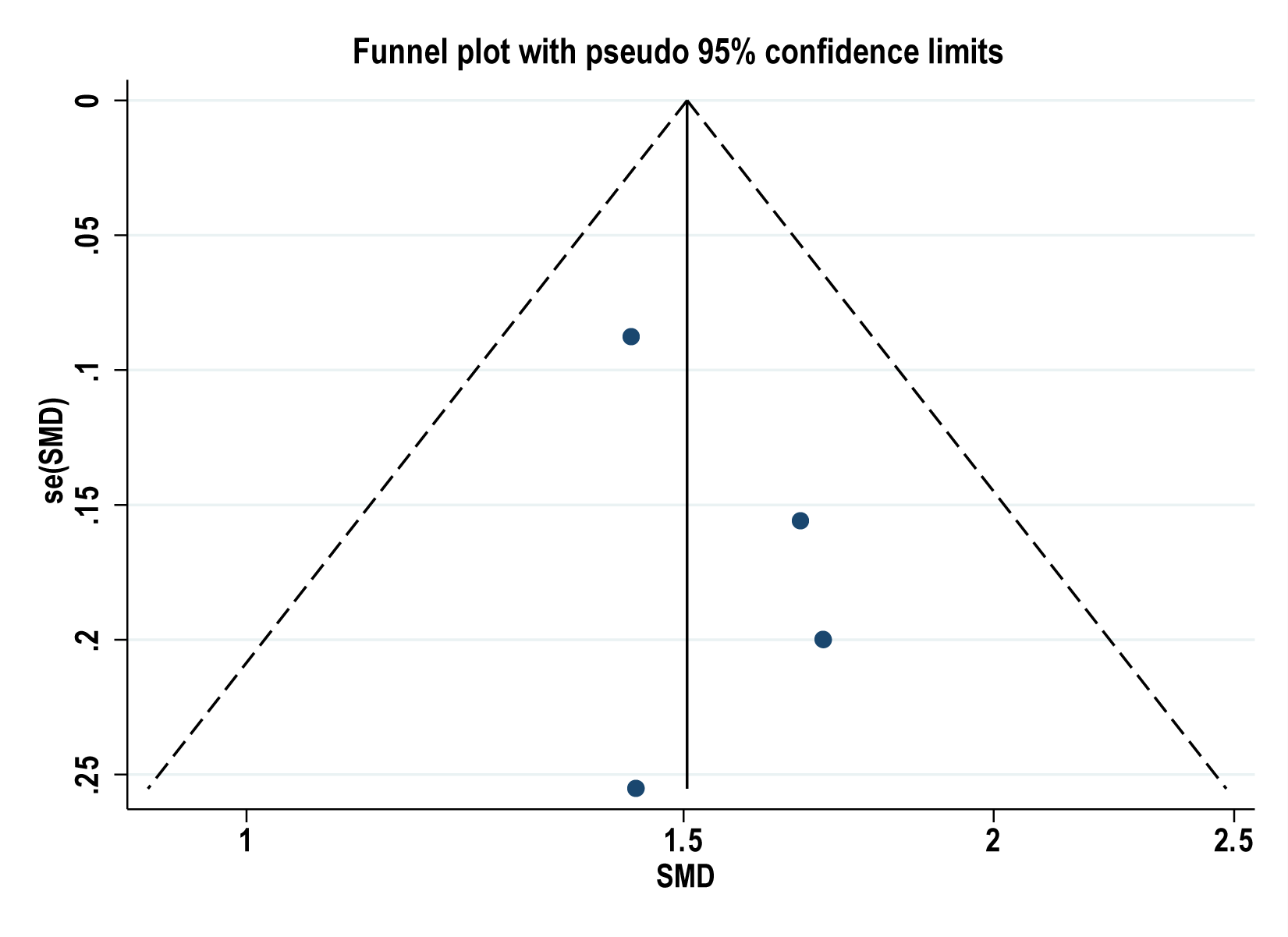

Supplement: Supplementary file 1 [file Datasheet1.zip › Data Sheet 1_v1/Supplementary Figure3B.tif]

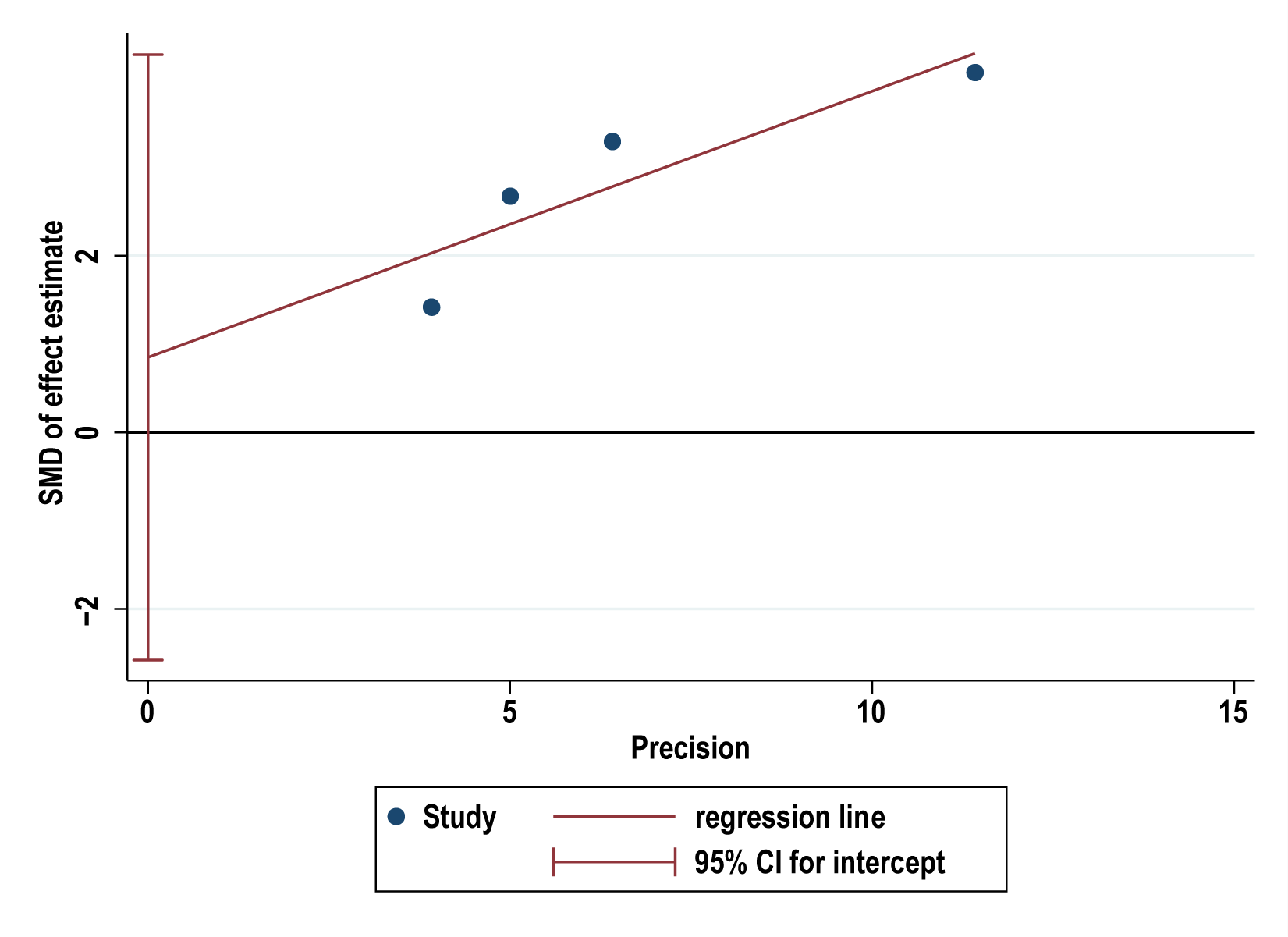

Supplement: Supplementary file 1 [file Datasheet1.zip › Data Sheet 1_v1/Supplementary Figure3C.tif]

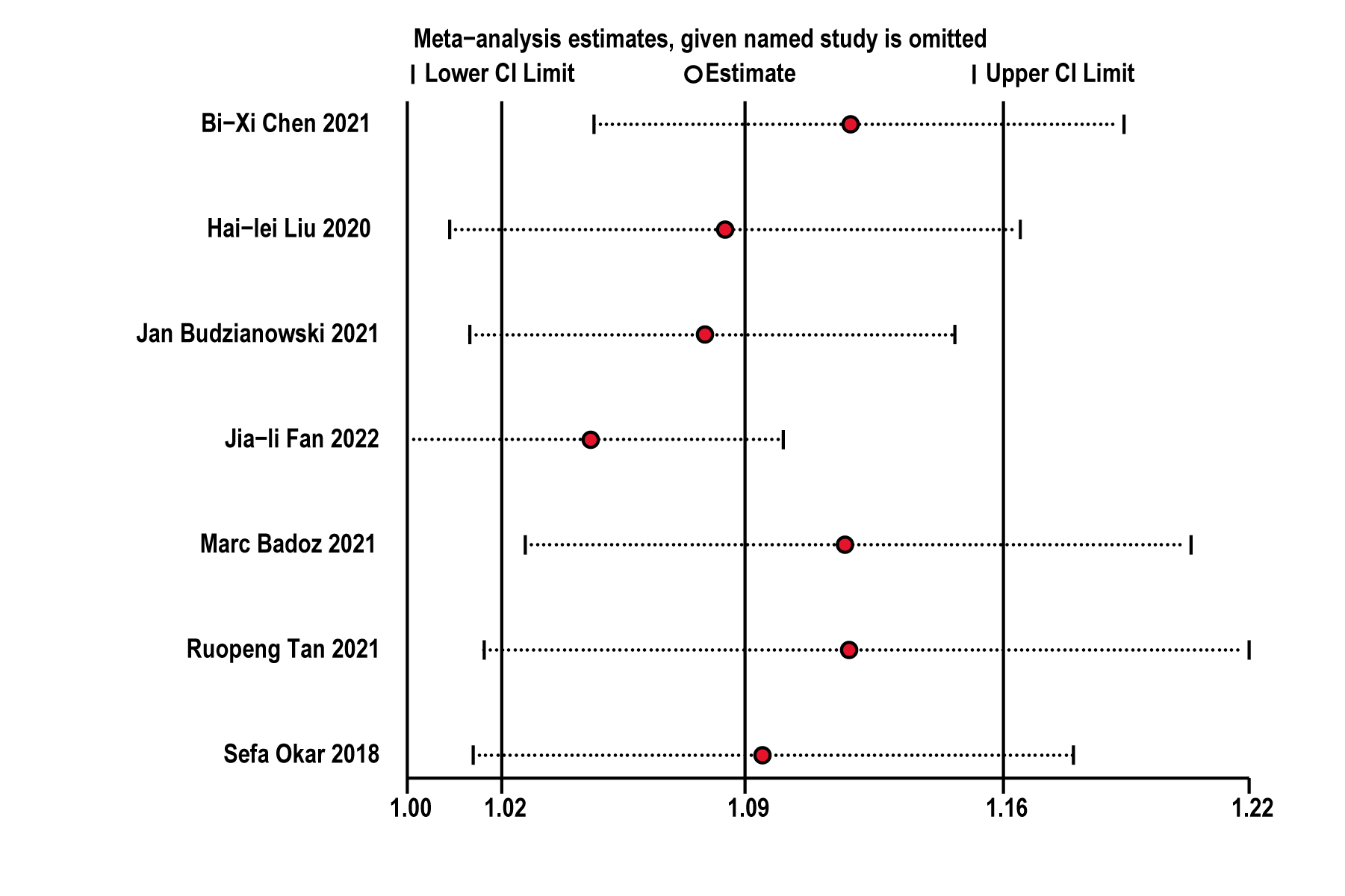

Supplement: Supplementary file 1 [file Datasheet1.zip › Data Sheet 1_v1/Supplementary Figure4A.tif]

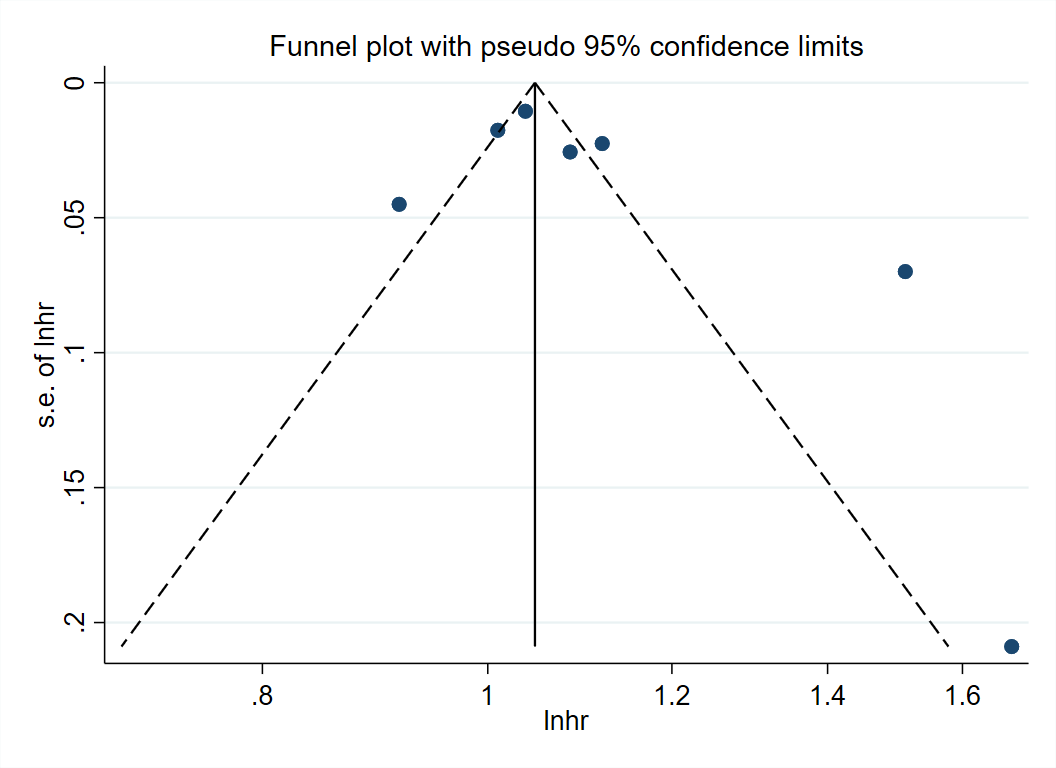

Supplement: Supplementary file 1 [file Datasheet1.zip › Data Sheet 1_v1/Supplementary Figure4B.tif]

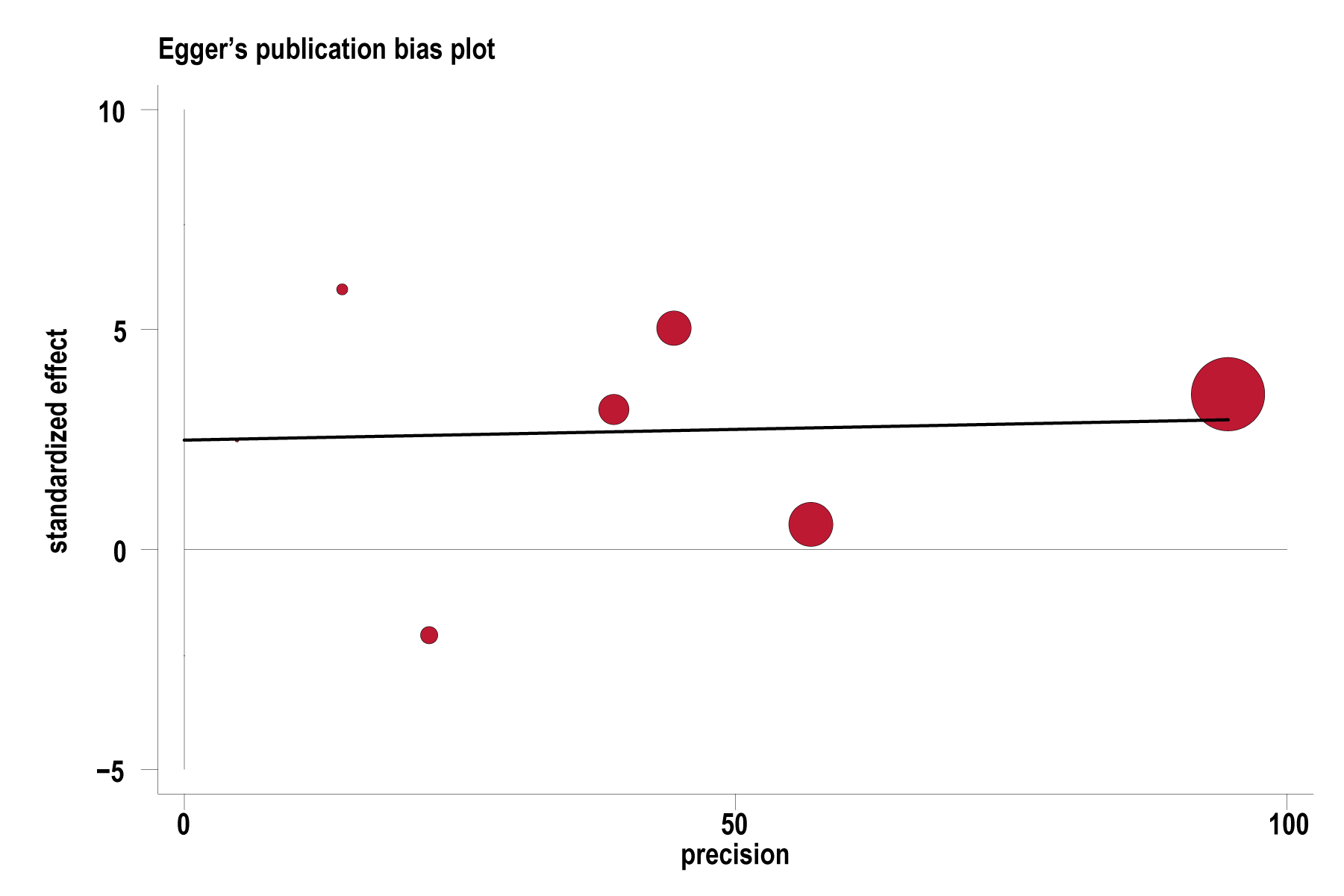

Supplement: Supplementary file 1 [file Datasheet1.zip › Data Sheet 1_v1/Supplementary Figure4C.tif]

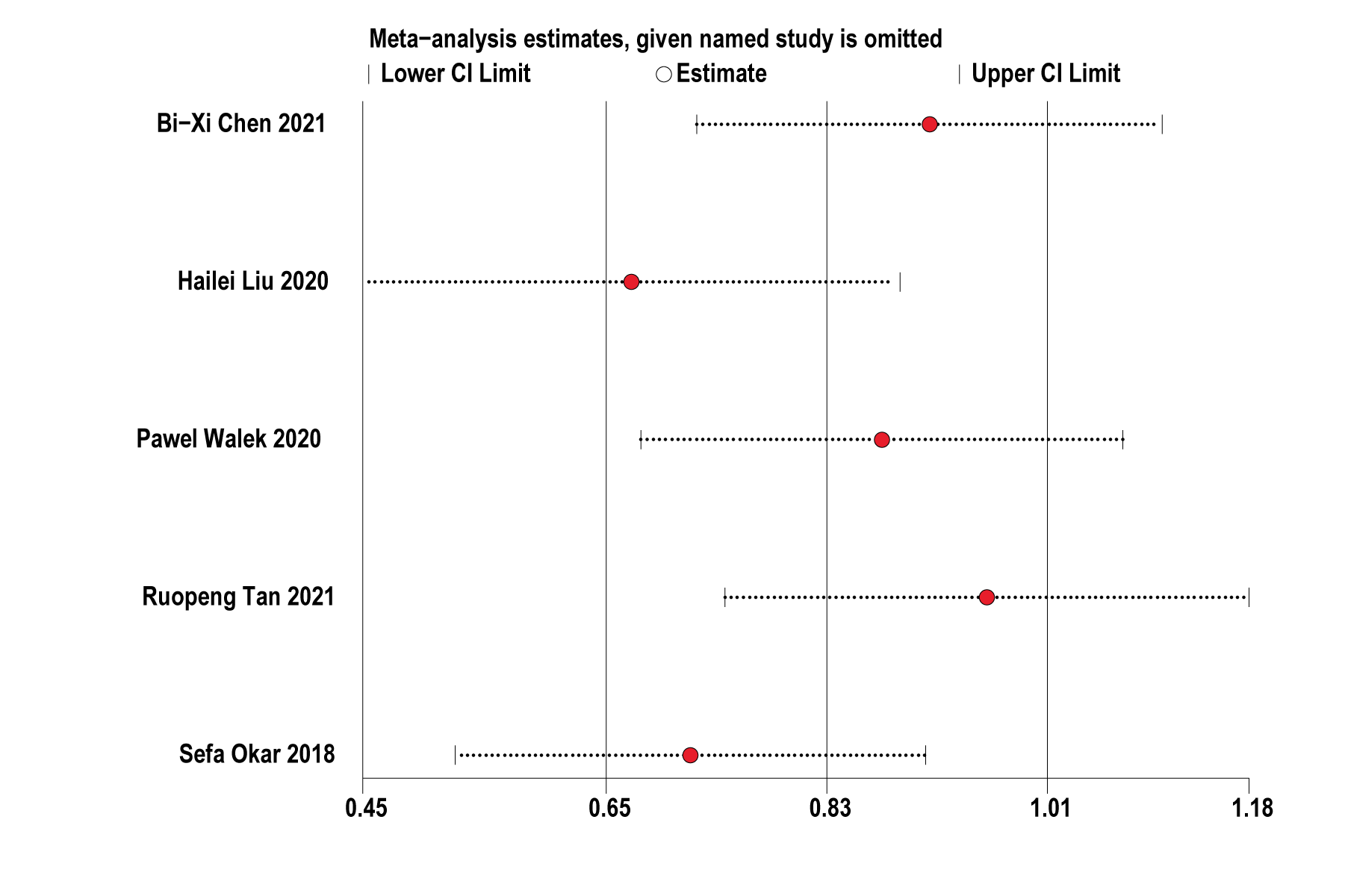

Supplement: Supplementary file 1 [file Datasheet1.zip › Data Sheet 1_v1/Supplementary Figure5A.tif]

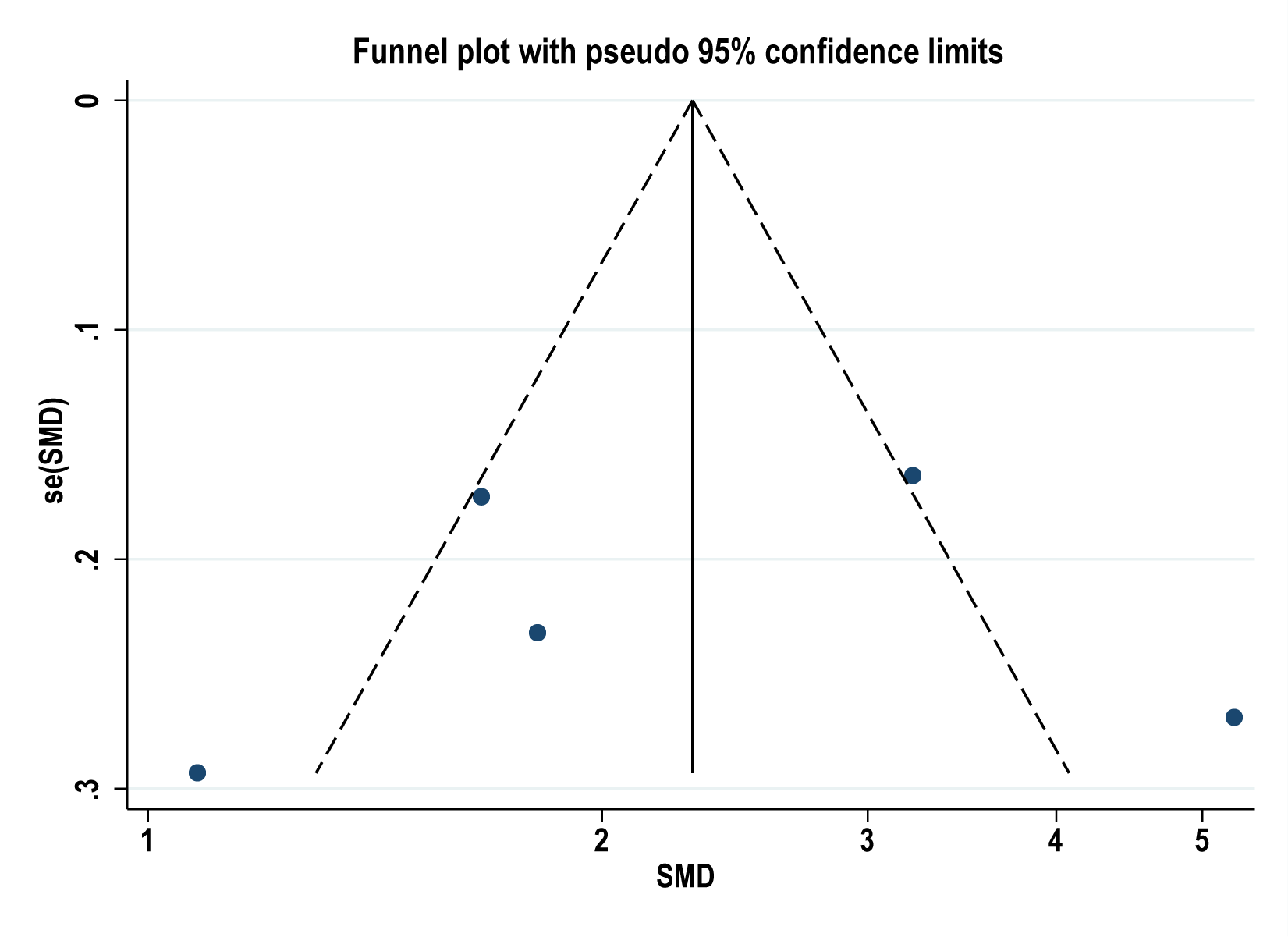

Supplement: Supplementary file 1 [file Datasheet1.zip › Data Sheet 1_v1/Supplementary Figure5B.tif]

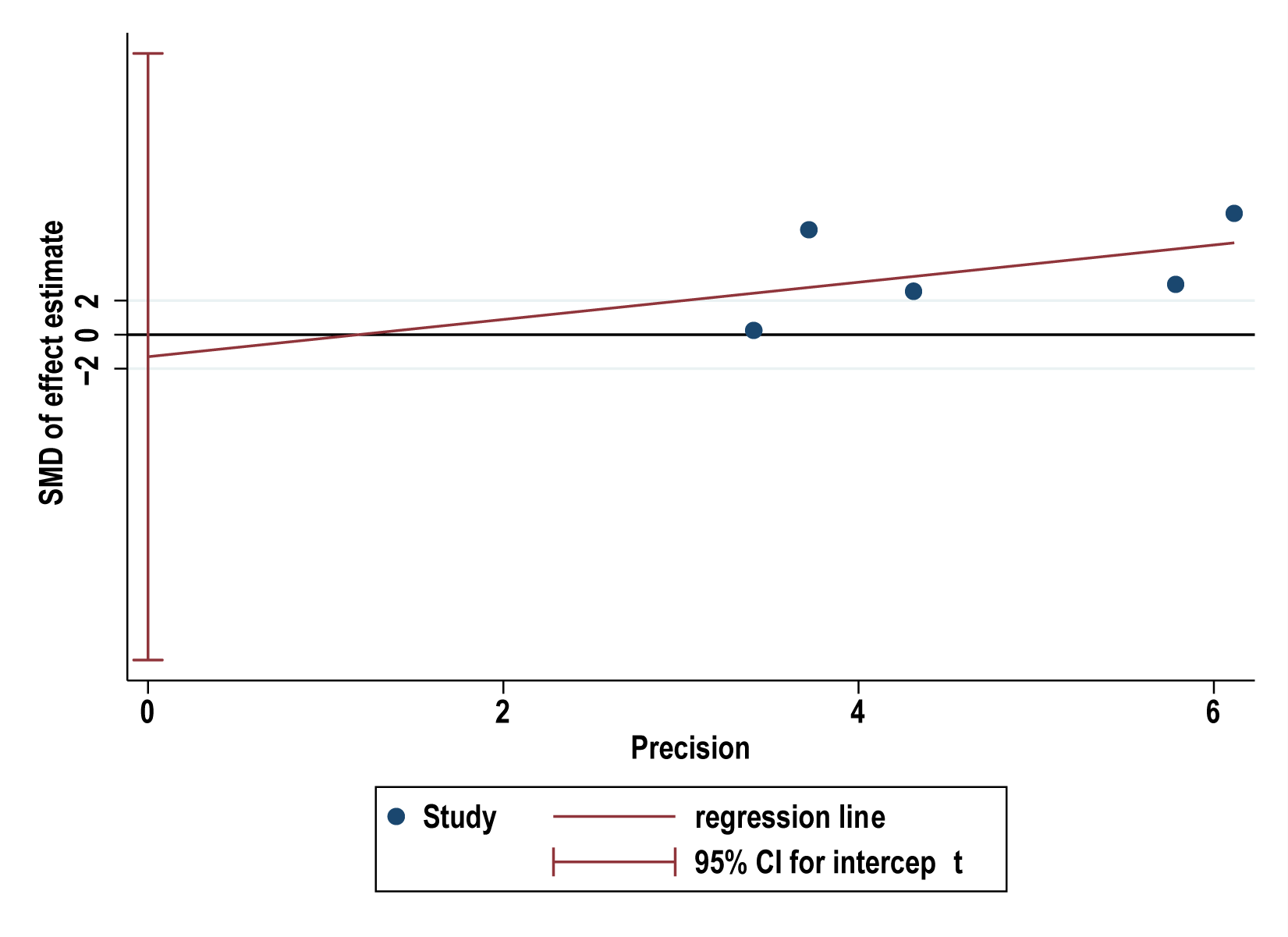

Supplement: Supplementary file 1 [file Datasheet1.zip › Data Sheet 1_v1/Supplementary Figure5C.tif]

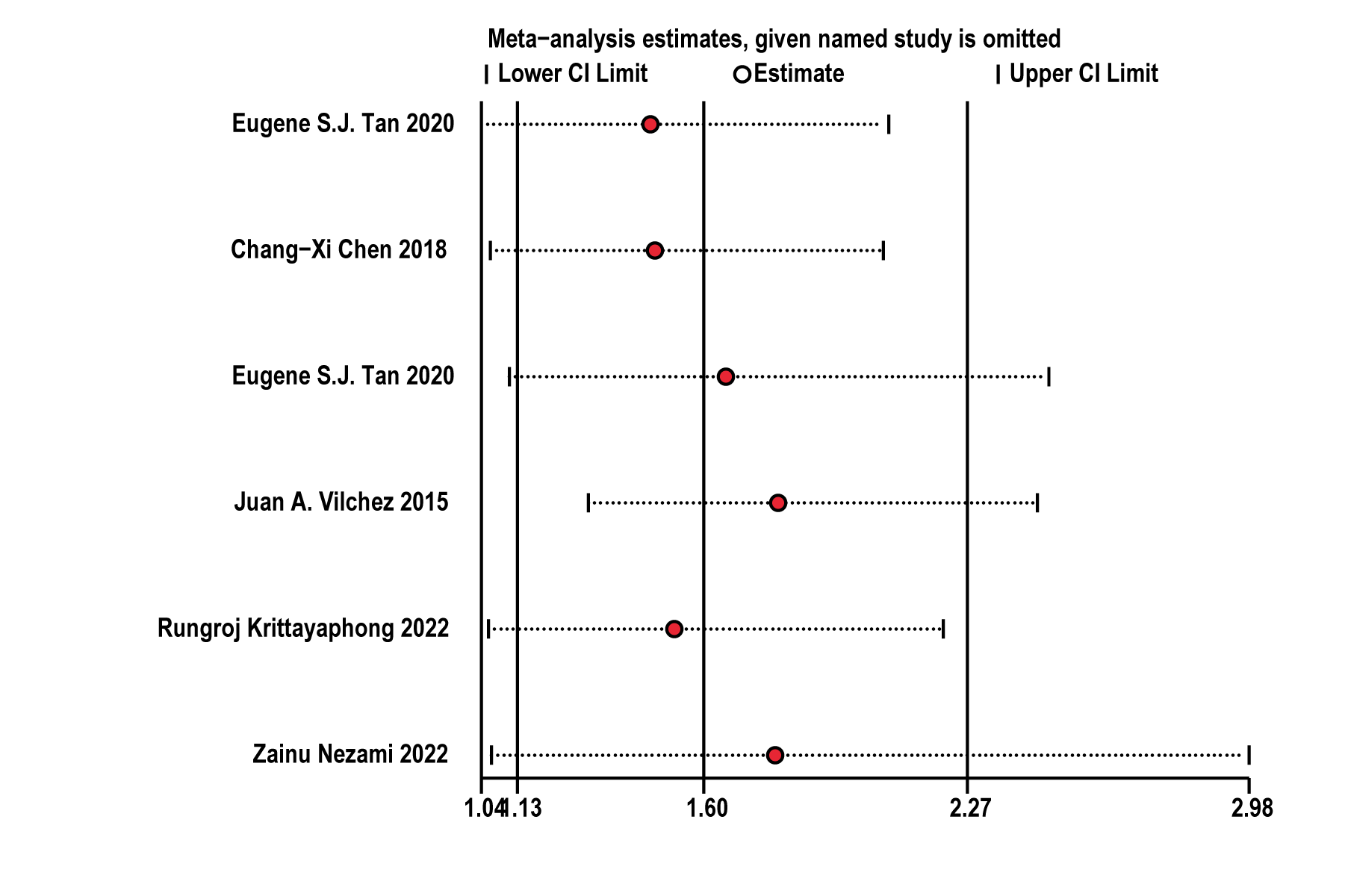

Supplement: Supplementary file 1 [file Datasheet1.zip › Data Sheet 1_v1/Supplementary Figure6A.tif]

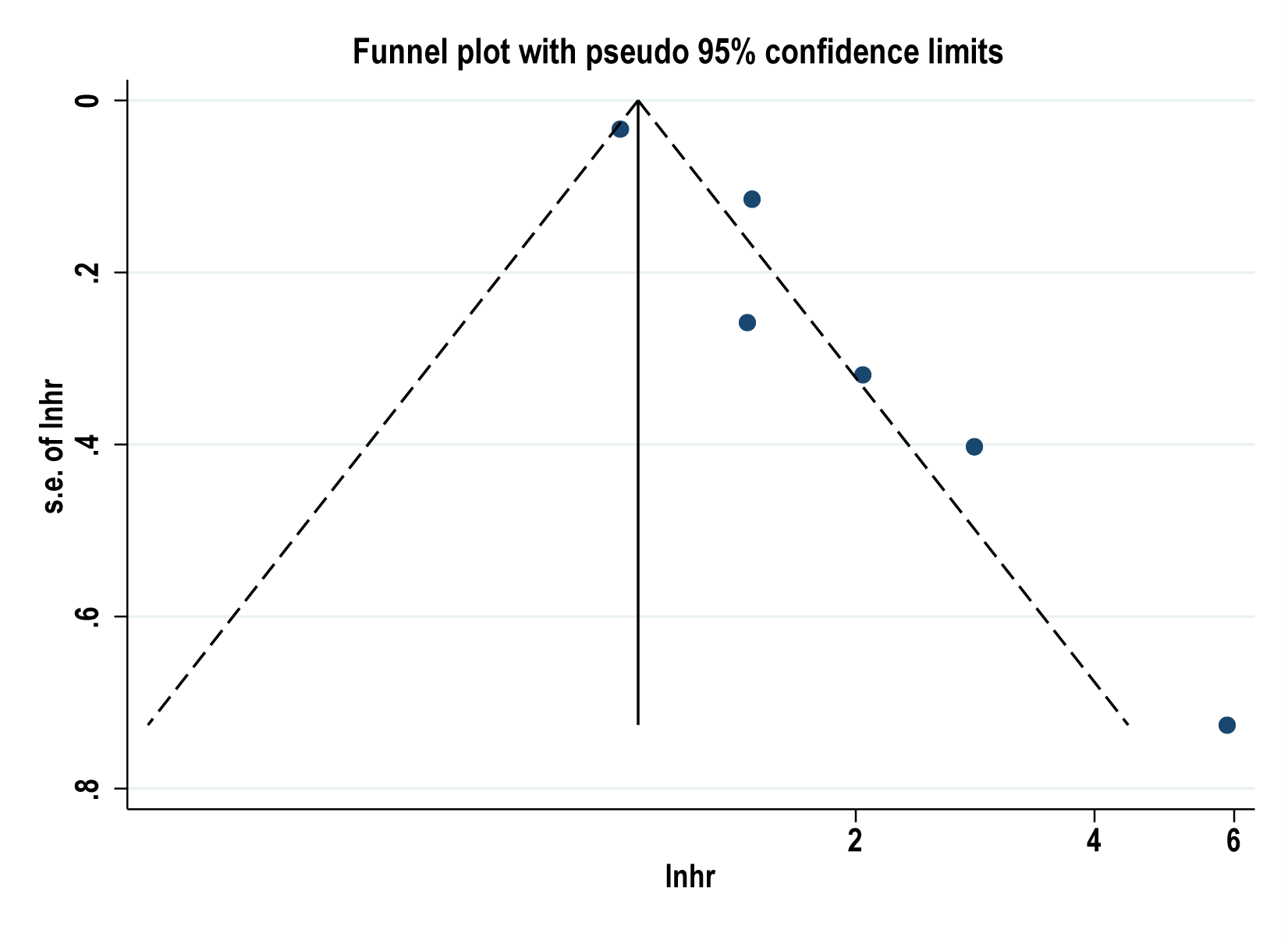

Supplement: Supplementary file 1 [file Datasheet1.zip › Data Sheet 1_v1/Supplementary Figure6B.tif]

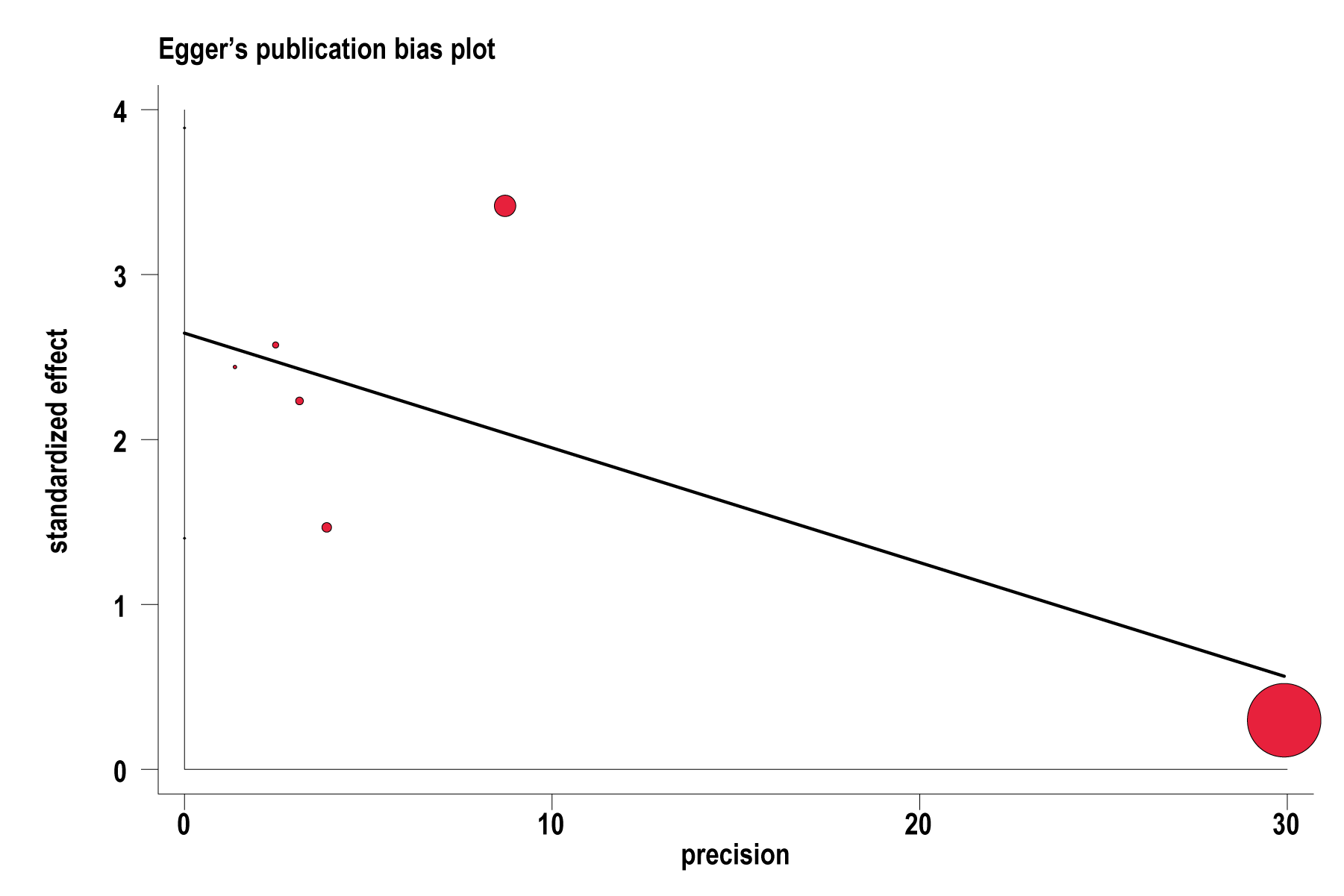

Supplement: Supplementary file 1 [file Datasheet1.zip › Data Sheet 1_v1/Supplementary Figure6C.tif]
