# Supplementary material for: Predictive value of soluble suppression of tumorigenicity 2 in atrial fibrillation: a systematic review and meta-analysis
Source: Front Cardiovasc Med. 2024 Jan 11;10:1308166. doi: 10.3389/fcvm.2023.1308166 (PMC10808625; doi:10.3389/fcvm.2023.1308166)
Supplement: Supplementary file 2 [file Datasheet2.doc]

**Supplementary Table1. Quality Assessment of the 24 icluded studies was assessed by the Newcastle-Ottawa scale**

| study（First,Author,Year） | Select | | | | Comparability | Outconme | | | Total |
| --- | --- | --- | --- | --- | --- | --- | --- | --- | --- |
|  | Exposed Cohort | Nonexposed Cohort | Ascertainment of Exposure | Outcome of Interest |  | Assessment of Outcome | Length of Follow-up | Adequacy of Follow-up |  |
| Ana Merino-Merin 2022 | * | * | * | * | * | * |  | * | 7 |
| Are A. Kalstad 2021 | * | * | * | * | * | * |  | * | 7 |
| Bi-Xi Chen 2021 | * | * | * | * | * | * | * | * | 8 |
| Chang-Xi Chen 2018 | * | * | * |  | * | * | * | * | 7 |
| Eugene S.J. Tan 2020 | * | * | * | * | * | * | * | * | 8 |
| Hai-lei Liu 2020 | * | * | * | * | * | * | * | * | 8 |
| Jan Budzianowski 2021 | * | * | * | * | ** | * | * | * | 9 |
| Jan-Thorben Sieweke 2020 | * | * | * | * | * | * |  | * | 7 |
| Jia-li Fan 2022 | * | * | * | * | * | * | * | * | 8 |
| Juan A. Vlchez 2015 | * | * | * | * | * | * | * | * | 8 |
| Julio A. Lamprea-Montealegre 2019 | * | * | * |  | * | * | * | * | 7 |
| Lei Chen 2022 | * | * | * |  | * | * |  | * | 6 |
| Marc Badoz 2021 | * | * | * | * | * | * | * | * | 8 |
| Michiel Rienstra 2015 | * | * | * | * | * | * | * | * | 8 |
| Nisha Bansal 2022 | * | * | * |  | * | * | * | * | 7 |
| Paweł Wałek 2020 | * | * | * | * | * | * | * | * | 8 |
| Rungroj Krittayaphong 2022 | * | * | * | * | * | * | * | * | 8 |
| Ruopeng Tan 2021 | * | * | * | * | * | * | * | * | 8 |
| Santeri Nortamo 2017 | * | * | * | * | * | * | * | * | 8 |
| Sefa Okar 2018 | * | * | * | * | ** | * | * | * | 9 |
| Wei-Ping Sun (a) 2022 | * | * | * |  | * | * |  | * | 6 |
| Wei-Ping Sun (b) 2022 | * | * | * | * | * | * |  | * | 7 |
| Xian-liang Yan 2022 | * | * | * | * | * | * |  | * | 7 |
| Zainu Nezami 2022 | * | * | * |  | * | * | * | * | 7 |

* represent stars used in the Newcastle Ottawa Scale.

**Supplementary Table2. AF outcomes and GRADE classification in meta-analysis of observational studies.**

| **№ of studies** | **Certainty assessment** | | | | | | **Effect** | | | **Certainty** | **Importance** |
| --- | --- | --- | --- | --- | --- | --- | --- | --- | --- | --- | --- |
|  | **Study design** | **Risk of bias** | **Inconsistency** | **Indirectness** | **Imprecision** | **Other considerations** | **№ of observer group** | **№ of control group** | **Relative (95% CI)** |  |  |
| The difference in sST2 levels between patients with and without AF | | | | | | | | | | | |
| 4 | observational studies | not serious^a^ | not serious^b^ | not serious^d^ | not serious^e^ | all plausible residual confounding would reduce the demonstrated effect^f^ | 347 | 1773 | SMD 0.41  (0.27 to 0.54) | ⨁⨁⨁◯ Mediate | crucial |
| The relationship between sST2 levels and the risk of AF occurrence | | | | | | | | | | | |
| 10 | observational studies | not serious^a^ | very serious^c^ | not serious^d^ | not serious^e^ | all plausible residual confounding would reduce the demonstrated effect^f^ | 1601 | 9880 | HR 1.04  (1.02 to 1.07) | ⨁◯◯◯ Very low | crucial |
| The difference in sST2 levels between AF patients with and without recurrence after CA | | | | | | | | | | | |
| 5 | observational studies | not serious^a^ | very serious^c^ | not serious^d^ | not serious^e^ | all plausible residual confounding would reduce the demonstrated effect^f^ strong association^g^ | 178 | 553 | SMD 0.81  (0.33 to 1.28) | ⨁⨁⨁◯ Mediate | crucial |
| The relationship between sST2 levels and the risk of AF recurrence after CA | | | | | | | | | | | |
| 7 | observational studies | not serious^a^ | very serious^c^ | not serious^d^ | not serious^e^ | all plausible residual confounding would reduce the demonstrated effect^f^ | 208 | 603 | HR 1.09  (1.02 to 1.16) | ⨁◯◯◯ Very low | crucial |
| The relationship between sST2 levels and the risk of the MACEs | | | | | | | | | | | |
| 5 | observational studies | not serious^a^ | very serious^c^ | not serious^d^ | not serious^e^ | all plausible residual confounding would reduce the demonstrated effect^f^ dose response gradient^h^ | 1221 | 2330 | HR 1.60  (1.13 to 2.27) | ⨁⨁◯◯ Low | crucial |

#### Explanations

1. Risk of bias by NOS was judged low for individual studies (see Supplementary Table1).upgraded
2. The heterogeneity was considered to represent low (I^2^=0%).upgraded
3. The substantial heterogeneity between studies was detected (I^2^ >50%).downgraded
4. Appropriate population generalizability and outcomes applicability.upgraded
5. Narrow 95% confifidence interval.upgraded
6. All included studies in this meta-analysis were observational studies, we cannot rule out that some residual factors may reduce the demonstrated effect.downgraded
7. The magnitude of the effect was large (SMD>0.5).upgraded
8. There was evidence of signifcant dose–response association (HR>1.50).upgraded

CI confdence interval, HR hazard ratio, SMD standardized mean difference, AF atrial fibrillation, CA catheter ablation, HF heart failure, MACEs major adverse cardiac events.
